# **Persistence mechanisms of Crohn's disease-associated adherent invasive** ***E**scherichia **coli*** **within macrophages**

**DOI:** 10.1080/19490976.2025.2587402

**Published:** 2025-11-25

**Authors:** Emma Bruder, Hosni Nedjar, Nicole Quenech'Du, Caroline Chevarin, Emilie Vazeille, Marie Granotier, Parul Singh, Anthony Buisson, Nicolas Barnich, Olivier Espéli

**Affiliations:** a CIRB – Collège de France, CNRS, INSERM, Université PSL, Paris, France; b Microbes, Intestin, Inflammation et Susceptibilité de l'Hôte, UMR Inserm/ Université Clermont Auvergne U1071, USC INRAE 1382, Clermont-Ferrand, France

**Keywords:** AIEC, Crohn's disease, intracellular bacterial communities, inflammation, bacterial persisters

## Abstract

Patients with Crohn's disease exhibit abnormal intestinal colonization by *Proteobacteria*, particularly adherent-invasive *Escherichia coli* (AIEC). These bacteria predominate in the mucus, adhere to epithelial cells, colonize them, and survive inside macrophages. We recently demonstrated that the AIEC strain LF82 adapts to phagolysosomal stress through a two-step process: initial replication arrest generating stress-tolerant persisters, followed by the resumption of replication, leading to the formation of intracellular bacterial communities (IBCs) embedded in a biofilm-like matrix. Given the significant genomic diversity among strains with the AIEC phenotype, we performed a comparative genomic and functional analysis of 13 AIEC isolates from Crohn's disease patients in France and Spain. Our results demonstrate that IBCs are replicative niches for all AIEC strains within THP-1 macrophages, yet their formation relies on distinct mechanisms, including variations in phagosome detoxification, biofilm architecture, and macrophage responses. Our study identifies a strong positive correlation between vacuole acidification and persister induction, which underlies the intracellular survival of the different strains. Furthermore, we revealed distinct AIEC dissemination strategies outside macrophages, potentially contributing to the propagation of inflammation in the human host. These findings highlight that research on pathogens and pathobionts with dynamic genomes should extend beyond classical bacterial models.

## GRAPHICAL ABSTRACT

**Figure:**
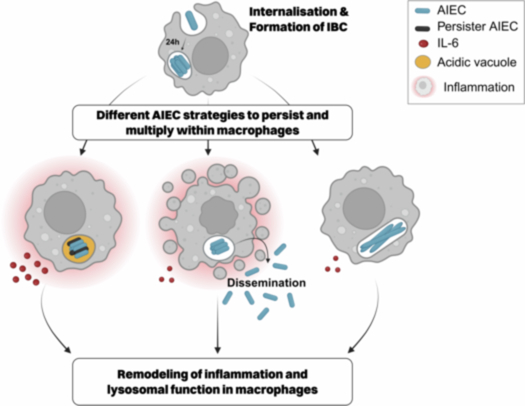

## Introduction

*E. coli* strains isolated from patients with inflammatory bowel disease (IBD) are frequently tested for a potential AIEC phenotype, i.e. adherence and invasion to epithelial cells and survival for 24 h within macrophages. The incidence of AIEC prevalence is particularly high in Crohn disease (CD) and ulcerative colitis (UC).^1^ These tests are standardized, and the attribution of the AIEC phenotype is based on quantitative indicators.^2^ As other intestinal pathogenic *E. coli* are extracellular pathogens, and intracellular multiplication is a particularly intriguing trait of AIEC.^3^^,^^4^ Recent animal model experiments have demonstrated that AIEC phenotype not only plays an ecological role in promoting inflammation^5^ but also contributes to disease etiology, such as intestinal fibrosis.^6^ Moreover, it has now been demonstrated that, compared to healthy individuals, macrophages in the intestines and *lamina propria* of CD patients harbor more bacteria. Among them, a large number of *Proteobacteria* were found in this dysbiotic microbiota.^6^^,^^7^ Such immune tolerance may contribute to the disease by sustaining inflammation. Hence, the necessity there is a clear need to better understand AIEC as a putative instigator or propagator of the disease is certain.

Only two clinically isolated AIEC strains have been used to describe molecular processes associated with epithelial cell and macrophage colonization: LF82, the first AIEC to be identified,^8^^,^^9^ and NRG857c.^10^ They belong to the B2 phylogroup of *E. coli* and are genetically very close. The current perception of LF82/NRG857c intra-macrophage lifestyle is that they reside in mature phagolysosomes and endure stresses brought by lysosomes, such as acidic pH, oxidative molecules, toxic compounds (metals or antimicrobial peptides) and nutrient depletion.^4^^,^^11^ Their eventual dissemination outside of this environment has not yet been described. We demonstrated that LF82 multiplication within macrophages results from a sophisticated strategy involving core genome responses, the activation of horizontally acquired pathogenicity islands, phenotype switches and the formation of intracellular communities within vacuoles.^12^^,^^13^ Interestingly, within macrophages, LF82 becomes antibiotic tolerant.^12^^,^^14^ The scenario of LF82 persistence differs from textbook's description of macrophage colonization by classical models such as *Salmonella*, *Brucella*, *Legionella* or *Mycobacteria*, which eventually counteract the immune response by secreting virulence factors.

AIEC identification is currently challenging because it relies on phenotypic assays based on infected cell cultures. To address this issue, a search for AIEC molecular markers has started; however, a specific and widely distributed genetic AIEC marker is still missing.^15^ Although different groups have analyzed the genome content of AIEC strains,^16^^,^^17^ only the complete and annotated genomes of three AIEC strains (LF82, NRG857c and UM146) are currently available in the NCBI database.^10^^,^^18^^,^^19^

Because intestinal colonization, adherence and invasion of epithelial cells are properties shared by *E. coli* pathogens, we postulated that intra-macrophage multiplication might be a genuine marker of AIEC. Therefore, in the present study, we combined genomic analyses of several AIEC strains with an in-depth characterization of their intra-macrophage persistence and multiplication strategies. We generated draft genomes from 10 newly isolated AIEC strains. We assembled and annotated the complete genomes of 4 new AIEC strains belonging to three different *E. coli* phylogroups (A, B1 and B2). We demonstrated that all AIEC form intravacuolar communities, which are characteristic of AIEC compared to other enterobacterial pathogens and commensals. Our study also showed that persistent communities can be formed using different pathways and may lead to different macrophage fates. Altogether, these data underscore the need to study more than one or few reference strains to understand host‒pathogen interactions, which is essential for developing effective approaches to fight infections. For bacterial strains with significant genomic plasticity, such as those in the ESKAPEE group, studying multiple strains is necessary to understand the mechanisms of infection.

## Materials and methods

### Strains and plasmids

The strains and plasmids used are described in Table S4. The AIEC strains analyzed in this study were isolated from ileal biopsies of Crohn's disease patients recruited in France between 2015 and 2016^20^ and from ileal or colonic biopsies of IBD patients enrolled in Spain between 2002 and 2007.^21^ In both cohorts, patients had not received antibiotics for at least two months prior to colonoscopy. The CEA224S ΔT3SS strain was created using the CRISPR method described in ref. [22]. The collection of new *E. coli* AIEC strains^20^ was sequenced by Illumina technology to generate draft genome contigs. For the strains CEA212U, CEA106S, CEA224S and CEA601S, genomic DNA was extracted with Invitrogen PureLink. Total DNA was sequenced by Nanopore technology (Novogen platform). To achieve *de novo* hybrid assembly of short and long reads passing quality control and filtering, we used the Unicycler v0.5.0 tool, with default parameters for chromosomes and plasmids.^23^

### Genome analysis and plasmid content

The genome annotation was performed with PROKKA v1.14.6 and based on LF82 annotation.^24^ *E. coli* phylogroup was determined with ClermonTyping,^25^^,^^26^ and the serotype was determined with ECTyper.^27^ Circos on plasmids sequence was performed using Galaxy.^28^^,^^29^ To identify antimicrobial resistance genes in chromosomes and plasmids, we used AMRFinderPlus version 3.11.26 with database version 2024-01-31.1. To search for virulence factors, we used VFDB on fasta assembly files, for TSS and defense systems, we used MacSyFinder and DefenseFinder/CRISPRCasFinder in MaGe.^30^^,^^31^ For prophages, we used Phaster and then Phigaro in MaGe. Finally, identification of T/AT systems was carried out with TAfinder2.0.^32^

### Pangenome analysis

To characterize the distribution of the gene content, a comparative analysis of the genomes of fifteen *E. coli* was performed using the pangenomic pipeline Anvi'o v7.1.^33^ The resulting pangenome was visualized in an interactive interface by running the script anvi-display-pan, where strains are clustered based on gene frequencies and the phylogram is clustered based on the presence/absence patterns of gene clusters. The pangenome tree including AIEC, commensal and pathogenic strains was constructed with GFF3 files provided by Prokka and used as input in Roary.^34^ A BLASTP identity threshold of 80% was applied, and a gene had to be present in 99% of the isolates to be considered part of the core genome. The tree was rooted with *Fergusonii* strains. Visualization and customization were performed with iTOL v6.^35^

### Infection

THP-1 (ATCC TIB-202) monocytes (4.75 × 10^5^ cells/mL) were differentiated into macrophages in phorbol 12-myristate 13-acetate (PMA, 20 ng/ml; Sigma-Aldrich, P1585). Infections were performed at MOI 100, with 120 µL of bacteria at OD600 = 0.5. The macrophages were centrifuged for 10 min at 900 rpm to activate phagocytosis and placed 10 min at 37 °C and 5% CO_2_. Then, the infected macrophages were washed twice with PBS, and a fresh RPMI medium containing 20 µg/mL gentamicin was added for the indicated durations (1 h to 72 h). This protocol leads to an effective MOI of 3 phagocyted bacteria per macrophage.

### Viable bacterial count using the gentamicin protection assay

The infected macrophages were then washed once with PBS, and 500 µL of 1% Triton X-100 in PBS was added to each well for 5 min to lyse the macrophage cells.^11^ The resulting samples were mixed, serially diluted, and plated onto LB agar plates to determine the number of colony-forming units (CFUs) recovered from the lysed macrophages.

### Antibiotic tolerance assay to measure persisters

Intracellular bacteria within THP-1 macrophages were extracted by lysing the host cells with 1% Triton X-100 in PBS. The lysates were centrifuged, and the bacterial pellet was resuspended in 500 µL of PBS. A 100 µL aliquot was used for bacterial enumeration through serial dilution and plating on LB agar plates. The remaining bacterial suspension was subjected to antibiotic stress by incubation with ciprofloxacin at 100x MIC (1 μg/ml) for 3 h at 37 °C, with shaking at 180 rpm. Following antibiotic exposure, the bacteria were serially diluted and plated onto LB agar plates to determine the number of viable colony-forming units (CFUs). To estimate the proportion of bacteria tolerant to antibiotic stress, the CFU count after the 3-h antibiotic treatment was normalized to the initial CFU count prior to antibiotic exposure.

### Immunolabelling and microscopy analysis

Infected THP-1 macrophages were fixed and immunostained by Lamp-I antibodies (DSHB, H4A3) and WGA (Thermo Fisher, W32466/W32464, or Biotium, 29027-1) staining was performed as described previously.^13^ Microscopy was performed as described previously^36^ on an inverted Zeiss Axio Imager with a spinning disk CSU W1 (Yokogawa).

### Quantification of IBC within macrophages

The number of bacteria within IBCs was quantified using Fiji software. Background fluorescence from the 488 nm channel was subtracted, and IBC structures were manually traced to measure the total fluorescence signal. The total fluorescence was then divided by the average fluorescence intensity of at least five individual bacteria, enabling an estimation of bacterial numbers within each community. At 1 h post-phagocytosis (P.P.), at least seven IBC structures were analyzed. Between 24 and 72 h P.P., when a higher number of IBCs were observed, between 66 and 305 structures were quantified, depending on the bacterial strains and experimental conditions.

### Intracellular pH detection of macrophages

To assess the macrophage pH, 30  min before imaging, the macrophages were treated with 0.5  nM pHrodo™ Red (Thermo Fisher, P35372) for 45  min at 37 °C and 5% CO_2_. The cells were rinsed 3 times with PBS and incubated in RPMI containing 20 µg/mL gentamicin. Images were acquired using a Spinning W1 Objective X40. Quantification was then performed using Fiji software, where red intensity was quantified in bacteria-infected macrophages and normalized by the red intensity of the bystanders.

### Live-cell imaging

All live-cell analyses were performed using Nunc Lab-Tek II Chambered Coverglass (Thermo Fisher, 155382). The acquisition was performed on an Axio Observer 7 Definite focus 2 video microscope (Carl Zeiss Micro-Imaging) equipped with a thermostatic chamber (37 °C, 5% CO_2_). Acquisitions were performed every 15 min for 16 h. Analysis was done using Fiji software.

### Bafilomycin assay

THP-1 macrophages were treated 30 min before infection with 150 nM of bafilomycin (Sigma Aldrich, B1793). THP-1 macrophages were then infected as described in the infection section, with the difference being that the treated macrophages were then put in contact with RPMI with 20 µg/mL of gentamicin and 150 nM of bafilomycin again. Microscopy, live bacteria counting and persisters measurement were performed as described above.

### Live-bacteria imaging

A time-lapse video was recorded to monitor the growth of the CEA212U strain on a LB-Agar pad. The overnight bacterial culture was diluted 1/1000 in LB-Agar. The growth process was observed using an Axio Observer 7 Definite Focus 2 video microscope (Carl Zeiss Micro-Imaging) equipped with a thermostatic chamber set at 37 °C. Images were acquired every 20 min for 3 h.

### FRAP analysis

FRAP was performed on THP-1 macrophages infected with LF82-GFP, CEA601S-GFP or CEA106S-GFP. Infections were conducted in fluorodish (World Precision Instruments). FRAP was performed at 48 h P.P. Acquisitions were taken every 10 s using a Plan-APO 60 × /1.4NA objective on a Ti Nikon microscope enclosed in a thermostatic chamber (Life Imaging Service) equipped with an Evolve EMCCD camera coupled to a Yokogawa CSU-X1 spinning disk. Four images were taken before photobleaching, and fluorescence recovery was followed every 10 s for 5 min. Fluorescence recovery was collected with Metamorph Software (Universal Imaging) and was analyzed using the Fiji Software.

### STED imaging

THP-1 macrophages infected by the different GFP strains were fixed and immunostained as described in Prudent et al.^13^ Imaging was performed with STED microscopy using an Axio Observer 7 (Carl Zeiss Micro-Imaging) equipped with a STEDYCON module (Abberior) using a ×100 oil-immersion objective. Detection was realized with avalanche photodiodes with filter cubes for green (500–550 nm), red (605–625 nm), and far red (650–720 nm) fluorescence.

### Macrophages activity

THP-1 macrophage viability was determined by colorimetric method using the CellTiter 96® AQueous One Solution Cell Proliferation Assay (Promega; G35C) as per the manufacturer's instructions. Briefly, 1 × 10^5^ cells were loaded into each well in a 96-well plate and infected as described previously. After the addition of 20 µL of reagent, the plates were incubated and measured using TECAN Spark microplate reader. All values were normalized and compared to standard curves.

### Western blot analysis

Cell extracts were obtained using a homemade Laemmli buffer devoid of reducing reagents (200 mmol/L Tris pH 6.8, 8% SDS, 40% glycerol, 0.2% bromophenol blue). SDS‒PAGE analyses were performed with 4%–12% NuPAGE bis-tris gels (Thermo Fisher Scientific, NP0336) and immunoblotted on nitrocellulose membranes (BioTrace Pall Laboratory, 732–3031) with antibodies: anti-LC3 (1/500; Sigma-Aldrich L8918), anti-actin (1/1000; Sigma-Aldrich, A2066), anti-NFkB (1/1000; Thermo Fisher Scientific, 51-0500), and anti-RpoB (1/1000; Biolegend, 663907). The secondary antibodies provided by Jackson Immuno-Research anti-Rabbit-HRP (1/5000; 711-035-152). Proteins were detected using SuperSignal West Pico or Femto (Thermo Fisher Scientific, 34580 or 34095), using Vilber Fusion-Fx (Vilber). Quantification was performed with Fiji software.

### Flow cytometry

Infected THP-1 macrophages were washed twice with PBS, and lysed for 5 min with 1% Triton X-100, filtered (40 µm; VWR, 732-2757) and fixed in formaldehyde (3.7% final) for 30 min. The samples were centrifuged, washed once and resuspended in PBS. The flow cytometry measurements were obtained with a CytoFlex LX cytometer and were managed using CytExpert. Analysis was performed using FlowJo Software.

### Recovery growth and CFU on supernatant

THP-1 macrophages were seeded at 4.75 × 10^5^ macrophages/mL, 2 mL/well and infected with AIEC culture at OD600 = 0.5 as described previously. After 48 h of infection, the macrophage supernatant was removed, and 1 mL of antibiotic-free RPMI medium was added after 2 PBS washes. 2 h later, the well supernatant was collected and centrifuged: 1). For the CFU count, 500 µl of this supernatant was removed before the cells were resuspended. Next, conventional CFU counts are performed with an easy-spiral-interscience machine (to enumerate live bacteria in the supernatant 2). For growth evaluation, 500 µl of this supernatant was removed before the addition of 500 µl of LB medium. Growth was measured for 16 h with Tecan Spark. The macrophages remaining in the wells were lysed with 1% Triton, and the CFUs were determined as previously described^13^ and counted with Scan300-Interscience software.

### Cytokines quantification

Infection in THP-1 macrophages was performed as described previously. The quantification of cytokines in the macrophages' supernatant was measured using MACSPlex Cytokine kit as per the manufacturer's protocol (Milteny, calibration: 130-106-197; TNF-α: 130-109-694; IL-6: 130-109-691; INF-γ: 130-109-695). Flow cytometry measurements were obtained with CytoFlex LX cytometer and were managed using CytExpert. Analysis was performed using Flowreada.io Software.

### Transcriptome analysis

Total RNA extraction in infected THP-1 macrophages was performed using TRIzol (Invitrogen, 15596018) protocol.^13^ The RNA libraries and sequencing processing were performed by Biomarker Technologies (BMKGENE), along with the subsequent analysis. The raw data are available on GEO under the reference GSE307944.

### qRT-qPCR

Total RNA bacterial extraction was performed on an overnight bacterial culture; the OD was adjusted to 0.2. RNA extraction was performed with AquaPhenol™ (MP Biomedicals, 11466960) protocol. Reverse transcriptase was carried out with iScript Reverse Transcription (Biorad, 1708891) after DNase treatment (Sigma, AMPD1-1KT DNaseI), and qPCR was conducted with iQ SYBR Green Supermix (Biorad, 1708880) on 1 µL of cDNA and 5 µM of primers. The data were acquired on Biorad CFX Connect Real-Time System in the Biorad CFX Maestro Software.

## Results

### Phylogenetically distant AIEC strains colonize macrophages

**Figure 1.**
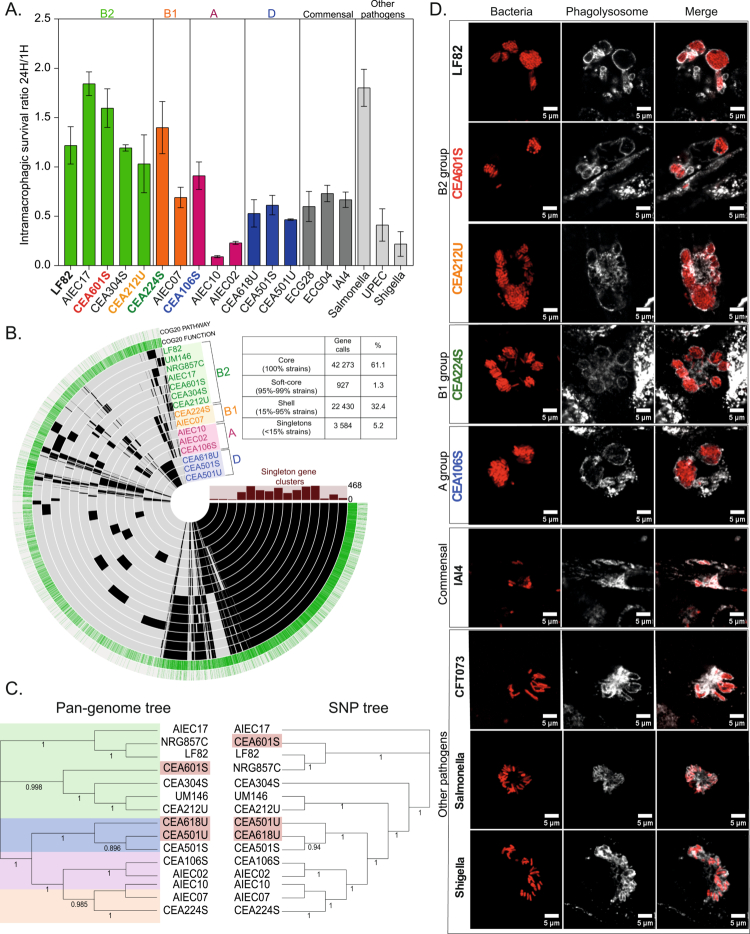
In spite of different genome contents, every AIEC colonize macrophage by forming IBC. A) Intramacrophagic survival ratio of 13 AIEC strains, 2 strains closely related to AIEC (ECG04 and ECG28), IAI4 (a commensal strain), and 3 pathogenic strains (*S. flexneri*, *S. typhimurium* LT2, and UPEC CFT073) within THP-1 macrophages over 24 h, compared to 1 h. B) Representation of the pangenome of 15 AIEC strains generated with Anvi'o. The layers correspond to individual genomes organized by their phylogenomic relationships. In these layers, black indicates the presence of a gene cluster, while lighter color signifies its absence. The colors of the labeled genes correspond to different phylogroups of *E. coli* (B2: green; B1: orange; A: pink; D: blue). C) Phylogenetic tree of 15 AIEC strains, which is based on pangenome genes (left) and SNPs (right), generated with Roary and Parsnp. The numbers on the branches represent the bootstrap values. D) Imaging of intracellular bacterial communities (IBCs) within Lamp-I-positive vacuoles in THP-1 macrophages formed by AIEC, commensal, and pathogenic strains at 24 h post-phagocytosis (P.P.). The scale bars are 5 µm.

To delineate strategies for persistence and multiplication within macrophages, we gathered *E. coli* strains exhibiting the AIEC phenotype (adhesion, invasion of epithelial cells and survival within macrophages, Figure 1A and Supplementary Figure S1A). These strains were collected from CD patient biopsies in the course of two different clinical multicenter studies.^20^^,^^21^ While draft genomes were previously available for some strains,^16^ we sequenced the remaining strains. Annotation of the assembled contigs unveiled significant genomic diversity within this group (Figure 1B and 1C). Core genes represented 61.1% of the pangenome (42273 gene calls), soft core genes accounted for 1.3%, shell genes for 32.4%, and singleton genes for 5.2%. Notably, certain strains harbor over 400 singleton gene clusters that are absent in all other strains. Consistent with this diversity, AIECs from our collection were assigned to four of the six *E. coli* phylogroups (A, B1, B2, and D).

To rationalize the comparative analysis of AIEC strains, we focused our efforts on characterizing their persistence and multiplication within a single host cell type, the THP-1 macrophage cell line, which offers the advantage of highly reproducible experimental conditions. We assessed the strains' capacity to persist in macrophages by quantifying colony-forming units (CFU) at 24 h post-phagocytosis (P.P.) (Figure 1A) and evaluating their ability to form intravacuolar communities (Figure 1D and Supplementary Figure S1B). For the majority of strains tested, macrophage survival is robust, but their multiplication is not extensive (with a 24-h to 1-h ratio comprised between 0.5 and 2). Notably, strains from the B2 group exhibit higher survival rates (median survival rate = 1.23) compared to those from the A (median survival rate = 0.62; *p*-value = 0.0002) and D (median survival rate = 0.70; *p*-value = 0.0007) groups. Only AIEC from the B2 and B1 groups showed 24-h survival rates comparable to those of *Salmonella Typhimurium* and significantly greater than those of the commensal strains. UPEC CFT073 strain and *Shigella flexneri* exhibited an artificial low survival rate caused by the massive death of macrophages (Figure 1A). In spite of their different survival dynamics, communities of bacteria residing within Lamp-I-positive vacuoles were consistently observed for each AIEC strain (as depicted in Figure 1D and Supplementary Figure S1B). These communities comprised tens to hundreds of bacteria densely packed within a single vacuole (Supplementary Figure S1C). Notably, a single macrophage can harbor multiple vacuoles containing a high density of AIECs. Interestingly, these AIEC communities exhibit stark differences from the phenotypes observed with the commensal *E. coli* IAI4, which rarely assembles into communities. Furthermore, this way of life in intravacuolar communities differs from that of other tested pathogens: the urinary tract infection CFT073, *Salmonella*, or *Shigella*. Although CFT073 and *Shigella* killed ~70% of the macrophage population before 24 h, the remaining macrophages presented respectively 85% and 82% of isolated bacteria in different compartments and very rare vacuoles with more than 6 bacteria. Macrophage infected by *Salmonella* are still alive after 24 h; they display rare communities with carrying capacities higher than 5 bacteria per vacuole (Figure 1D). These observations underscore the significance of intravacuolar community formation as a defining characteristic of AIECs, despite their diverse genomic contents.

### In depth analysis of the genome content of five different AIECs

**Figure 2.**
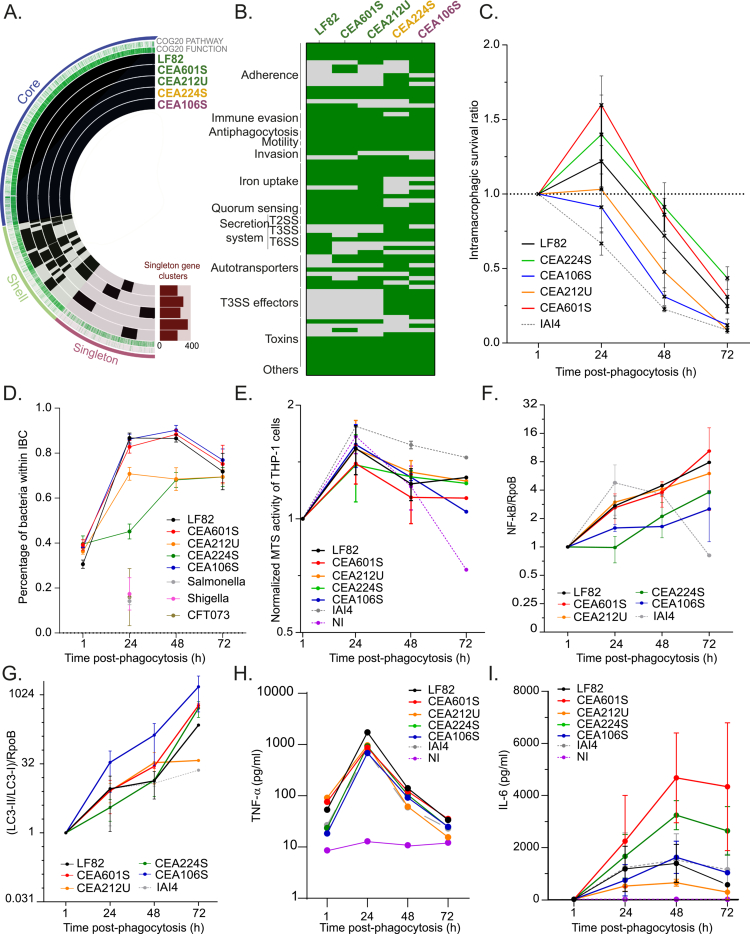
Analysis of the virulence arsenal and macrophage response to AIEC strains. A) Presence/absence chart of the genomes of the 5 AIEC strains studied in the following figures generated with Anvi'o. The layers represent individual genomes organized by their phylogenomic relationships. In the layers, black indicates the presence of a gene cluster, while light color indicates its absence. The detailed analysis of the presence and absence of gene clusters is presented in Supplementary Figure 3B) Evaluation of the virulence arsenal of the 5 AIEC strains. Green indicates the presence of a gene, and gray indicates its absence. C) Survival of AIEC strains within macrophages during a 72-h infection kinetics. Error bars represent the mean and standard error of the mean (SEM); *n* ≥ 3. Individual data points are shown in Supplementary Figure 7B for each strain and in Supplementary Figure 7C for each time point. The data were analyzed using the Mann‒Whitney test: *p < 0.05, **p < 0.01, ***p < 0.001, ****p < 0.0001. D) Quantification of bacteria involved in intracellular bacterial communities (IBCs) relative to the total bacteria in the population. The error bars represent the mean and SEM. E) MTS cell activity assay of THP-1 macrophages during the infection kinetics with AIEC strains. The error bars represent the mean and SEM. F) Western blot quantification of the inflammatory response in infected macrophages, as measured by NF-κB levels normalized to *E. coli* RNA polymerase (RpoB) levels and relative to the 1-h post-phagocytosis (P.P.) values. The error bars represent the mean and SEM. G) Western blot quantification of the autophagy response in infected macrophages, as indicated by the LC3II/LC3I ratio normalized to the RpoB level and relative to the 1-h P.P. The error bars represent the mean and SEM. H) TNF-*α* quantification in the culture media of macrophages infected by AIEC or the commensal strain IAI4. I) IL-6 quantification in the culture media of macrophages infected by AIEC or the commensal strain IAI4. The error bars represent the mean and standard deviation (SD).

To refine our understanding of AIEC intramacrophage persistence and multiplication, we curated a subset of four new AIECs spanning the B1, B2, and A phylogroups. By employing a combination of Illumina and Nanopore sequencing techniques, we assembled the complete genomes of each strain (Figure 2A and Supplementary Tables S1 and S2, see Methods). Notably, we achieved a single contig for the chromosome of each strain and identified several plasmids ranging from 1.5 kb to 133 kb (as illustrated in Supplementary Figure S2A and S2B). Large plasmids found in strains CEA601S, CEA212U, CEA224S, and CEA106S exhibit significant homologies with the NRG857c and UM146 plasmids (Supplementary Figure S2C), yet show no similarity with the Cyrano phage plasmid from LF82.^10^^,^^19^^,^^37^ Even within this limited bacterial cohort, genomic diversity remains striking, with nearly one-third of the genome comprising shell or singleton genes (prophages, phage defense systems, toxin‒antitoxin systems, and metabolic islands exhibiting notable disparities (Supplementary Figure S3). None of the prophages hosted by LF82 were identified in the other four strains. Instead, they possess different complete or incomplete prophages, possibly inserted at analogous genomic loci. While some of these prophages may carry virulence factors, such as the Shiga toxin in PhiP27-like prophage or GtrII-GtrA proteins in phage SfII, meticulous examination revealed that none of these virulence factors were actually encoded by the prophages within our study group (see Supplementary Table S2).

The four newly identified AIECs manifest highly diverse pathogenicity gene profiles (Figure 2B, Supplementary text and Supplementary Figure S3 A−G)^38–42^. Even strains from the B2 group (CEA601S and CEA212U) exhibit divergent sets of fimbriae, invasion factors, agglutinins, colicins, iron capture systems, and secretion systems compared to LF82. Interestingly, CEA224S, the strain from the B1 group that efficiently colonizes macrophages (Figure 1A), harbors on its plasmid a putative type three secretion system (T3SS) and type 3 effectors similar to *Salmonella enterica* SPI-1.^43^ Deletion of the CEA224S's T3SS showed a reduction in intra-macrophage survival, suggesting that AIEC might obtain extra weapons from horizontal gene transfer (Supplementary Figure S3H). Our detailed genomic description suggests that the LF82 strategy may not be universally adopted among other members of our study group.

### Virulence of AIECs within macrophages

We assessed bacterial viability over a 72-h period post-phagocytosis. An equal amount of exponentially growing AIEC strains and IAI4 commensal strains were exposed for 20 min to THP-1 macrophages differentiated from monocytes. This resulted in a phagocytosis rate of 1 to 10 bacteria per macrophage for LF82, CEA601S, CEA212U, and CEA106S, while a slightly higher rate was observed for CEA224S 1 h after phagocytosis. About 80% of the macrophages were infected with LF82 and CEA224S while only half of them were infected with the other strains at the 1-h time point. Interestingly, even at this early time point, about 5%–10% of the infected macrophages presented vacuoles containing more than 3 bacteria (Supplementary Figure S1C).

For LF82, CEA224S, and CEA601S, the proportion of viable bacteria remained relatively stable for 48 h before decreasing to 0.3–0.5 at 72 h (Figure 2C). In contrast, the numbers of CEA106S and CEA212U declined more rapidly, suggesting less efficient survival or more frequent macrophage death.

Throughout this infection kinetics, the majority of AIEC bacteria resided within packed communities containing up to hundreds of bacteria and were delimited by a membrane decorated by Lamp-I, hereafter called IBC. The frequency of AIEC within IBC ranged from 60 to 94% between 24 and 72 h post infection, depending on the strain (Figure 2D and Supplementary Figure S1C). Even strains with low colony-forming units (CFU) at 72 h, such as CEA106S and CEA212U, exhibited IBC frequencies exceeding 70% (Figure 2D). These IBCs started as small communities at 1 h and increased in size, reaching a maximum of 392 bacteria per one IBC for CEA106S at 48 h post-phagocytosis (median = 22.6; IQR = 38.5; Range = 389.2) (Supplementary Figure S1C). In contrast, the IBC detected at 24 h post-phagocytosis for *Shigella*, *Salmonella* and CFT073 contained less than 20% of the bacterial population (Figure 2D). Thus, IBC appears to be the preferred lifestyle of AIECs within macrophages despite survival and genomic variabilities.

To investigate the impact of IBC on macrophages, we assessed metabolic activity, cell viability, inflammation, autophagy, and cytokine production. We utilized formazan to monitor ATP synthesis in infected macrophages (Figure 2E), which remained stable throughout the infection and was comparable to non-infected controls. This suggests that macrophage viability was not significantly affected by infection and therefore did not explain the decline in the bacterial population at the 72 h mark. We quantified NF-κB as a marker of inflammation and LC3-II for autophagy. Consistent with the existing literature, strains LF82, CEA601S, CEA212U, and our commensal control IAI4 induced inflammation (Figure 2F), with approximately 4 times more NF-κB in macrophages at 48 h post-phagocytosis. Surprisingly, for strains CEA106S and CEA224S, we observed only 1.6- and 2 times more NF-κB, respectively, at 48 h post infection. Additionally, all strains promptly induced LC3-II conversion, a marker of autophagy (Figure 2G). Infection with CEA106S stood out with a much more rapid and potent induction of autophagy. The presence of bacteria in macrophages promote an important secretion of TNF-α compared to uninfected macrophages at 24 h post-phagocytosis. However, the TNF-α levels significantly decreased at 48 h for all strains (Figure 2H). Finally, measurement of IL-6 production by macrophages shows a high amount of IL-6 for the CEA601S and CEA224S strains, with an increase over time (Figure 2I). LF82 and CEA106S promoted less IL-6 secretion, CEA212U promoted even less but nevertheless a higher amount than the not infected control. These physiological features show poor correlations with bacterial survival, suggesting that strain specificity rather than bacterial load is the main driver of the macrophage response.

### Transcriptomic macrophage response to AIEC persistence

**Figure 3.**
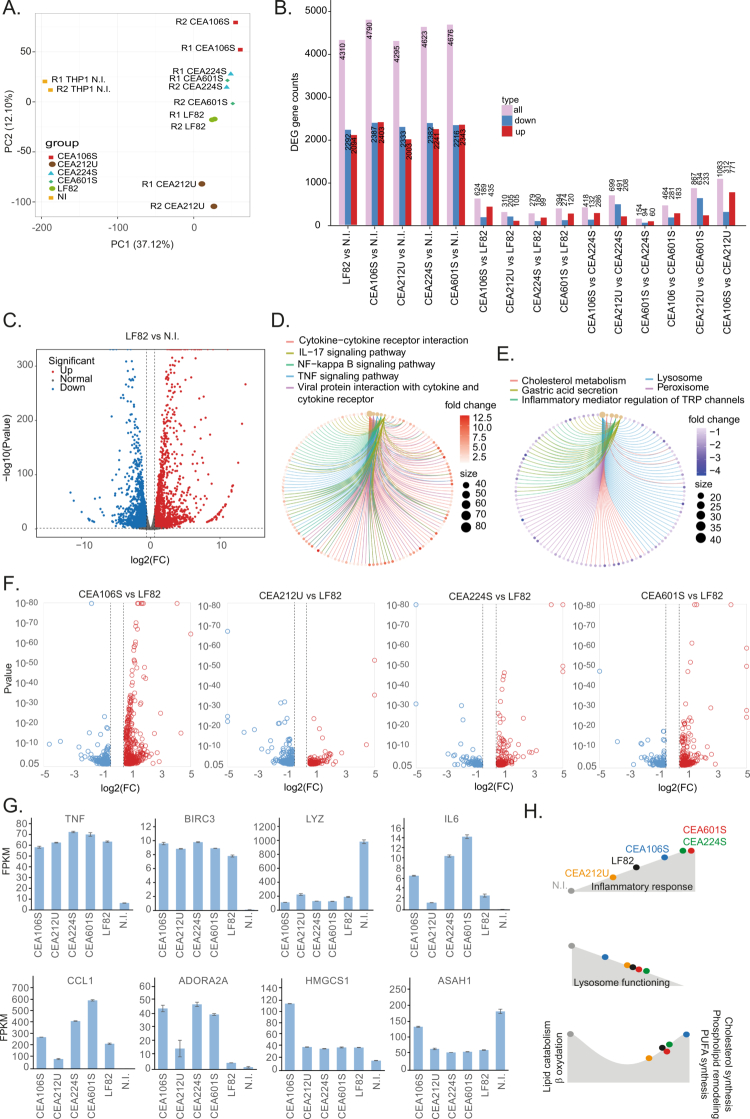
Transcriptome of infected macrophages. A) Principal component analysis of the transcriptome of control macrophages (N.I.) and those infected by five different AIEC strains. B) Number of differentially expressed genes (DEG) within different comparison couples. C) Volcano plot representing the massive transcriptome changes observed when LF82 persisted Compared to non-infected macrophages. D) KEGG analysis of the main upregulated pathways in the presence of LF82. E) KEGG analysis of the main downregulated pathways in the presence of LF82. F) Volcano plot representing the transcriptome changes observed when macrophages phagocytosed LF82 compared to the four other AIEC strains. G) Examples of genes exhibiting noticeable expression changes according to the phagocytosed strain. H) Illustration of the three main pathways that are differently expressed when different AIEC persisted in macrophages.

We used RNA-seq to assess the molecular consequences of the persistence of the five different AIEC strains. Principal component analysis of the macrophage transcriptomes 24 h after phagocytosis revealed a clear separation from the uninfected controls. Notably, replicates of the different experimental groups clustered apart along PC2 (Figure 3A). The transcriptional responses induced by strains CEA212U and CEA106S differed the most, whereas LF82, CEA601S, and CEA224S grouped more closely together. AIEC persistence altered the expression of approximately 4000 genes (Figure 3B, Table S3). For LF82, the magnitude of these changes was striking, highlighting a major rewiring of macrophage physiology (Figure 3C) toward a strongly inflammatory state driven by NF-κB, JAK–STAT, and TNF signaling pathways (Figure 3D, Supplementary Figure S4A). In parallel, AIEC persistence consistently downregulated genes involved in lysosome, peroxisome, and phagosome biogenesis and dynamics (Figure 3E, Supplementary Figure S5A). This downregulation is reminiscent of the early repression of lysosomal functions during *Salmonella* infection, which is mediated by the SopB effector acting on TFEB expression. However, since AIEC strains lack SopB homologs and do not inhibit TFEB expression (Supplementary Figure S5C), we propose that a distinct mechanism is responsible. Consistent with previous studies, despite this transcriptional repression of lysosomal genes, LF82 predominantly resides within Lamp-I-positive acidic compartments, underlining the originality of AIEC biology. Pathway analysis further identified ferroptosis signatures in infected macrophages (Supplementary Figure S4A). Examination of apoptosis- and ferroptosis-related genes revealed that, across all strains, prodeath signals were upregulated (e.g., TNF/BID/TRAIL for apoptosis; TFRC/NCOA4/ZIPs for ferroptosis), while the execution machinery was suppressed (caspases/APAF1/DFFB for apoptosis; ALOX15/LPCAT3 for ferroptosis). In parallel, protective regulators were induced (BIRC3, CFLAR, and MCL1 for apoptosis; SLC7A11 and GCLM for ferroptosis) (Figure 3G, Table S3). Thus, both cell death programs appear to be “primed but restrained” at 24 h post-phagocytosis, which is consistent with MTS assay results (Figure 2E).

Taken together, the common transcriptional response observed across the five AIEC strains suggests a host–pathogen “stalemate” strategy. Macrophages become hyperinflammatory and secrete chemokines and cytokines, yet remain viable because of the inhibition of apoptosis and ferroptosis. At the same time, bacterial clearance is impaired: antimicrobial phagolysosomal effectors (acidification, granule enzymes, lysozyme, DNASE2) and antigen presentation machinery (MHC-I, MHC-II) are significantly repressed. In the context of Crohn's disease, such a strategy may sustain local inflammation by recruiting additional immune cells while preventing bacterial eradication. This creates an environment where AIEC can persist and replicate within vacuoles for extended periods without host cell death or vacuolar rupture.

### AIECs specifically manipulate inflammatory response, lysosome biogenesis and lipid metabolism

Given that macrophages engage shared pathways in their response to AIEC, we next asked whether strain-specific differences (Figure 2; NF-κB activation, IL-6 secretion, and autophagy signaling) result from fine-tuning of these general responses. Comparing infections revealed between 154 and 1083 differentially expressed genes depending on the strain (Figure 3B and 3F, Supplementary Figure S6). Analysis of cytokine and interleukin genes showed an intriguing partition: CEA106S, CEA224S, and CEA601S induced higher expression of IL-1B, IL-6, IL-8, and CCL1, whereas CEA212U and LF82 induced more IL-18, an anti-inflammatory cytokine (Figure 3G, Supplementary Figure S4B). This cytokine pattern correlated with the expression of the adenosine A2A receptor (ADORA2A), a known regulator of macrophage polarization (Figure 3G, Table S3). Unexpectedly, ADORA2A was most strongly induced in infections associated with higher inflammatory responses (CEA106S, CEA224S, and CEA601S), the opposite of what is typically observed during *Salmonella* infection, where ADORA2A promotes an “M2” anti-inflammatory phenotype.^44^ Although ADORA2A expression does not necessarily imply receptor activation, this inversion further highlights the distinctiveness of AIEC–macrophage interactions.

Differences also emerged in lysosome-, peroxisome-, and phagosome-related genes. Macrophages infected with CEA106S were separated from the other strains in PCA analysis (Supplementary Figure S5B), as several genes in this category were not repressed and, in some cases, were overexpressed (e.g., ASAH, Figure 3G). The overexpression of ASAH, CTSB, TFRC, and NPC1 may help establish a distinct phagosomal niche for CEA106S, where acidification and proteolysis are partially preserved. Another major strain-specific feature was lipid metabolism. While genes associated with lipid catabolism and *β*-oxidation were generally repressed, genes involved in cholesterol (HMGCS1, Figure 3G), terpenoid (FADS1, Supplementary Figure S5C), and PUFA biosynthesis were upregulated, suggesting a switch favoring membrane biogenesis. Again, CEA106S stood out, with 27–35 lipid metabolism genes significantly more expressed compared to the other strains (Figure 3G, Supplementary Figures S5 and S6).

Overall, our transcriptomic analyses revealed that AIEC strains elicit both shared and strain-specific responses in macrophages. These differences affect inflammatory signaling, lysosomal aggressiveness, and lipid metabolism (Figure 3H) and may have profound consequences for bacterial persistence and for the physiology of infected tissues. However, a limitation of our study is that the THP-1 cell line is not a perfect mimic of human intestinal macrophages; therefore, these observations need further confirmation with primary cells or *in vivo* assays.

### Adaptation of AIECs within phagolysosomes

**Figure 4.**
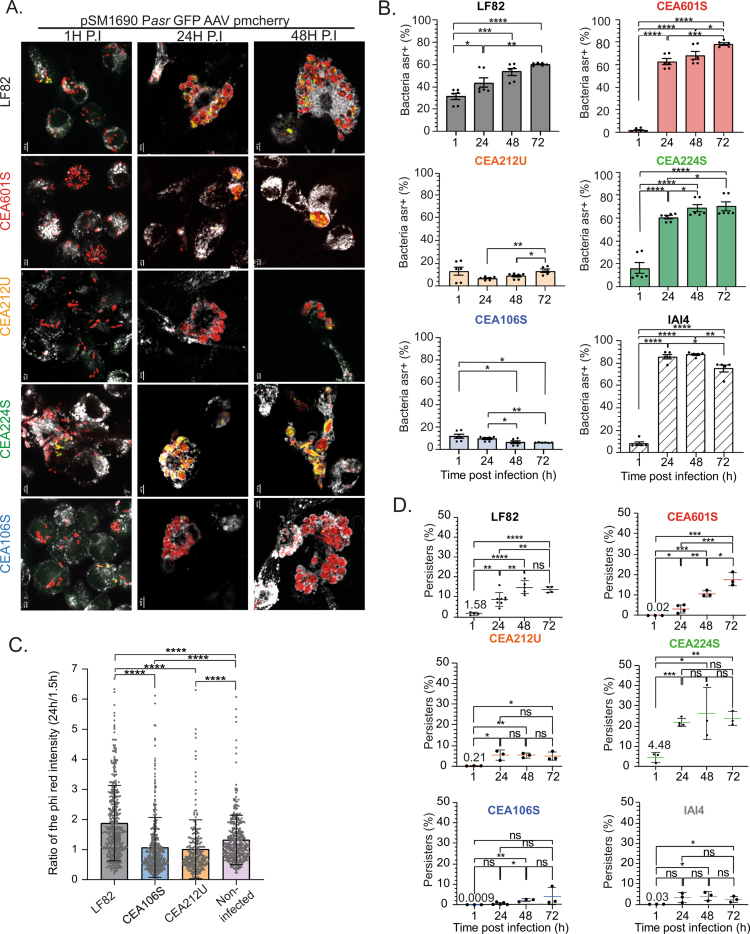
Characteristics of IBC formed by different AIECs. A) Imaging of THP-1 macrophages infected with AIEC strains carrying an unstable GFP reporter plasmid for *asr* expression (acid response). Imaging was performed at 1, 24, and 48 h post-phagocytosis (P.I.). Bacteria are shown in red, with green indicating activation of the *asr* promoter. Phagolysosomes are labeled with Lamp-I antibody and appear in gray. Scale bars represent 5 µm. B) Flow cytometry quantification of AIEC response to phagosomal acid stress (*asr* promoter). *p*-values are indicated (Student's *t*-test: *p < 0.05, **p < 0.01, ***p < 0.001, ****p < 0.0001); error bars represent the mean and standard error of the mean (SEM); *n* ≥ 3. C) Quantification of the intensity of the pHrodo probe in THP-1 macrophages infected by AIEC strains LF82, CEA106S, CEA212U, or uninfected. Each point represents the intensity ratio at 24 h compared to the mean intensity at 1.5 h post-phagocytosis in bystander THP-1 macrophages. *p*-values are indicated (Mann‒Whitney test: *p < 0.05, **p < 0.01, ***p < 0.001, ****p < 0.0001); error bars represent the mean and standard deviation (SD); n ≥ 3. D) Measurement of the proportion of persisters formed during AIEC growth within macrophages. *p*-values are indicated (Student's t-test: *p < 0.05, **p < 0.01, ***p < 0.001, ****p < 0.0001); error bars represent the mean and standard deviation (SD), *n* ≥ 3.

We hypothesized that variation of macrophage responses may reflect different AIEC adaptation strategies. We recently demonstrated that within macrophages, the LF82 transcriptome was dominated by responses to lysosomal stresses (acid pH, oxidative stress, alteration of the bacterial envelope.^12^^,^^13^ Since CEA106S infection differed from other AIECs in higher autophagy (Figure 2G), lysosome biogenesis and lipid metabolism (Figure 3H) capacities, we considered that the phagosomal niches might change according to the bacterial strain. We tested the acid response of the five strains with a biosensor based on the *asr* promoter fused with an unstable GFP (Supplementary Figure S7A). Asr-GFP imaging (Figure 4A) and flow cytometry analysis of lysed macrophages (Figure 4B and Supplementary Figure S7B) revealed two types of behaviors: strains LF82, CEA601S, CEA224S and IAI4 exhibited frequent and strong expression of the *asr* promoter; in contrast, strains CEA212U and CEA106S rarely exhibited expression of *asr* promoter. RT‒qPCR confirmed this observation; at 24 h post-phagocytosis, CEA106S and CEA212U had lower *asr* gene transcription compared to other AIEC strains (Supplementary Figure S7C). These observations fit with the lower inflammatory profile observed for CEA212U but are counterintuitive with the higher autophagy and lysosome capacities observed earlier for CEA106S (Figures 2 and 3); therefore, we directly checked macrophage acidification capacities. Both CEA106S and CEA212U reside in macrophages in a less acidic environment than LF82 and are nearly similar to noninfected macrophages (Figure 4C). Within macrophages, acidic conditions are used by pathogens as signals for various phenotypic switches, including the formation of persisters.^45^ Persisters correspond to a subset of the bacterial population that tolerates antibiotic treatment above MIC for a long period. Persisters exhibit low energetic metabolism and are therefore frequently called dormant bacteria. In fact, persisters are only a portion of the dormant bacteria group.^14^ We measured the frequency of persisters at 1, 24, 48 and 72 h P.P. for each strain. CEA601S and CEA224S followed the same trend as the one observed for LF82,^12^ persisters frequency increased during infection, reaching ~20% of the population. In contrast, CEA212U and CEA106S followed the commensal IAI4 trend, exhibiting very low and nearly constant level of persisters (Figure 4D). This high correlation between phagosomal acidification, the bacterial response to acidification and the persister frequency demonstrated that AIEC and macrophage physiology are tightly coupled.

### AIECs' way of life within phagosomes

The good correlation between persister frequency and *asr* induction prompted us to check whether the multiplication rates differed. We used a biosensor constructed with the *rrnBP1* promoter to monitor the stringent response. The five AIECs and IAI4 behave similarly, with only a small proportion of the bacteria (5%–15%) exhibiting high *rrnBP1* expression and therefore high translation capacity (Supplementary Figure S8A).

We labeled bacteria with a FtsZ-GFP reporter for cell division, which confirmed that fewer than 30% of the bacteria of each strain were attempting to divide at late time points (Supplementary Figure S8B and S8C). This relationship between FtsZ ring assembly and multiplication was altered for the strain CEA212U, which forms transient giant filaments that may divide into single cells populating IBC at late time points post-phagocytosis (Supplementary Figure S8D and Movie S1). This filamentation was not observed in LB medium (Figure S8E), confirming that, within phagosomes, CEA212U encounters a different environment compared to other AIEC. Finally, we investigated whether intracellular AIEC are planktonic or adherent in biofilm-like structures. We recently described that within IBCs, LF82 IBCs were structured by a matrix and can be considered microbiofilm structures.^13^ Surprisingly, only LF82 and CEA106S exhibit strong WGA labeling. In contrast, the strain CEA224S exhibited weak WGA labelling visible in some of the IBCs, and strains CEA601S and CEA212U did not exhibit any WGA labeling (Supplementary Figure S9A and S9B). This finding is in good agreement with the different movements of the bacteria within IBC (Supplementary Figure S9C). IBCs are therefore of different natures: AIECs either multiply within biofilm-like structures or in less rigid environments, where their movements are limited only by adherence between bacteria or between bacteria and the phagolysosomal membrane.

### A positive correlation between inflammatory response, persister frequencies and AIEC survival

**Figure 5.**
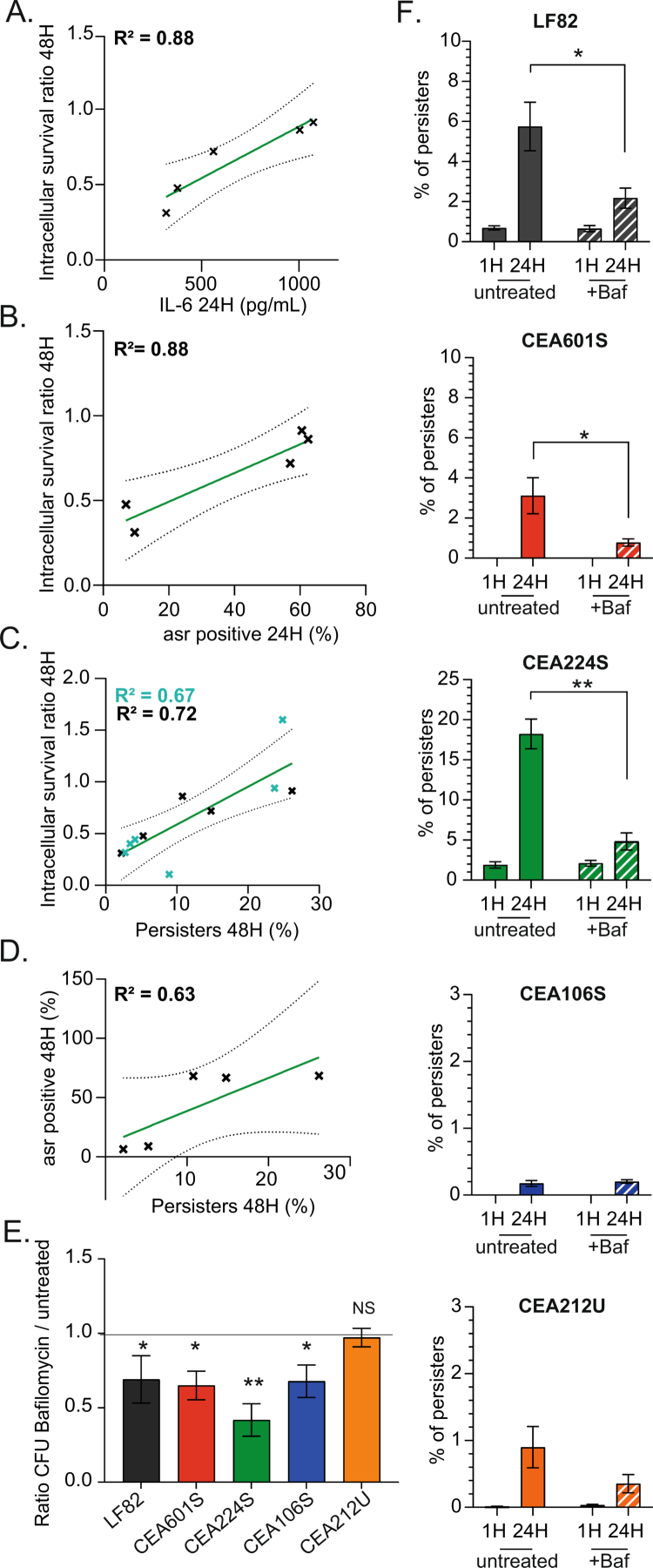
Correlation between the intracellular survival rate, acidic response and formation of persisters. A) Linear regression between intracellular survival (CFU from the gentamycin protection assay shown in Figure 2C) and IL-6 production secreted by infected macrophages (quantification shown in Figure 2I). The linear regression is depicted in green, with confidence bands represented by black dashed lines. B) Linear regression between intracellular survival and AIEC acidic response at 24 h post-phagocytosis (P.P.). C) Linear regression between intracellular survival and persister formation at 48 h P.P. The coefficient of determination (*R*²) is displayed in black for the 5 AIEC strains and in cyan when including five additional AIEC strains whose values are indicated by the cyan points. D) Linear regression between the AIEC acidic response at 24-h P.P. and persister formation at 48-h P.P. E) Survival ratio of the AIEC strains in THP-1 macrophages treated with BAF A1 compared to the survival ratio in untreated THP-1 macrophages. The error bars represent the mean and standard error of the mean (SEM), *n* ≥ 3. F) Quantification of persisters in THP-1 macrophages (filled bar graph) compared to persisters formed in BAF A1-treated THP-1 macrophages (dashed bar graph). *p*-values indicated the difference (Student's *t*-test compared to a hypothetical value of 1: *p < 0.05, **p < 0.01); error bars represent the mean and standard error of the mean (SEM); *n* ≥ 3.

In the course of this study, we collected about 60 phenotypic infection characteristics that helped us decipher AIEC intramacrophage persistence, multiplication and dissemination properties. To identify key phenotypes, we quantitatively analyzed the correlation of these phenotypes with AIEC survival (CFU). Three particular phenotypes strikingly correlate with survival: the production of IL-6 (*R*^2^ > 0.8), the frequency of persisters (*R*^2^ > 0.7) and the acid response of the bacteria (*R*^2^ > 0.8) (Figure 5A−D, Supplementary Figure S10A). These findings suggest that these three characteristics are connected. Interestingly, the frequency of persisters and the intracellular survival were still correlated when we integrated values from other strains from the AIEC collection that we did not characterize extensively (Figure 5C). To challenge the robustness of this correlation, we treated infected macrophages with bafilomycin A1 (BAF A1). BAF A1 inhibited the fusion of lysosomes with phagolysosomes and therefore curb acidification (Supplementary Figure S7D). We observed a significant reduction of the survival and persister frequencies of the strains usually contained in acidic vacuoles: LF82, CEA601S and CEA224S, but no effect on CEA212U, which did not exhibit acidification. This confirmed the relationship between acidification, persisters and survival, but again, CEA106S stood out with an intermediary phenotype involving a reduction of the survival but no effect on persister frequency (Figure 5E and 5F). These observations suggest that AIECs deal with the macrophage inflammatory response to colonize them more efficiently. Surprisingly, we did not observe a similar correlation with NF-κB and TNF-*α* production. Although more strains will be required to validate these observations, they open the way to simpler screening of AIEC collections.

### Fates of AIECs and infected macrophages

**Figure 6.**
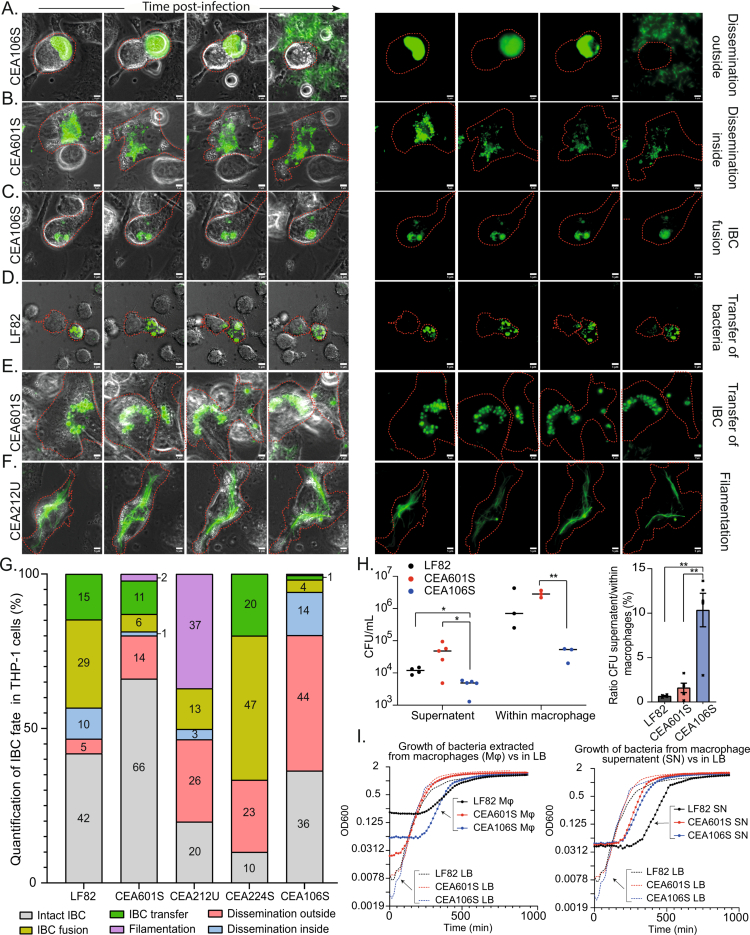
Fates of AIECs and macrophages Montage of live imaging of THP-1 macrophages infected by AIEC at 48 h post infection (P.P). Bacteria are shown in green, and macrophages are outlined in red. Scale bars represent 5 µm. Full movies are presented as supplementary materials. A) Illustration of the ejection of an IBC outside a macrophage. B) Illustration of the dissemination of an IBC within a macrophage. C) Illustration of IBC fusion. D) Illustration of the transfer of IBC bacteria from a donor to a recipient macrophage. E) Illustration of the transfer of an IBC from a donor to a recipient macrophage. F) Illustration of IBC filamentation. G) Quantification of the different IBC fates observed (A–F). H) Proportion of viable bacteria at 24 h P.P. in the supernatant of macrophages (gentamycin was removed for 2 h before collection) or within macrophages. The graph on the right represents the ratio of bacteria in the supernatant compared to bacteria within the macrophages. *P*-values are indicated (Student's t-test: *p < 0.05, **p < 0.01, ***p < 0.001, ****p < 0.0001); *n* ≥ 3. I) Recovery of bacterial growth following macrophage lysis (left) or from macrophage supernatant (right), compared to growth recovery from the stationary phase in LB medium (dashed line).

The dissemination of AIEC outside macrophages has not yet been documented. To assess whether IBC formed by AIEC within macrophages represent a dead end that may not benefit bacteria or a springboard for propagation, we monitored infected macrophages for 12 to 16 h at the 48-h time point. Frequent imaging at low laser intensity enabled us to track moving macrophages accurately and detect rapid events while preserving macrophage and bacterial viability. We observed hundreds of infected macrophages for each strain (Figure 6A–F and Movies S2–S13). Direct ejection of bacteria outside of dying macrophage was observed for every strain. However, in the case of LF82, CEA212U, and CEA106S, we also observed vacuolar rupture and dissemination within the cytosol of the macrophages. Additionally, every strain exhibited vacuole fusions, with these fusions being particularly frequent with CEA224S. More surprisingly, we observed the transfer of intact IBC from one macrophage to another for LF82, CEA601S, and CEA224S; most of the time, this transfer occurred concomitantly with the death of the donor macrophage, but it was also observed between live macrophages, resembling a process similar to vomocytosis. It is noteworthy that inter-macrophage transfers occurred in the presence of gentamicin in the medium, which did not seem to affect IBC integrity. For CEA212U, at 48 h P.P. many elongated bacteria (filaments) were observed, in the following hours, they either continue to grow or resolve, forming clonal IBC. Therefore, CEA212U filamentation did not directly facilitate dissemination as it does for UPEC in bladder cells. We measured the viability of the released bacteria in a 2-h window without gentamicin. LF82 and CEA601S exhibited a release rate of 1% of the total population in 2 h, while CEA106S exhibited a much higher release rate of 10% in 2 h (Figure 6H). Optical density measurements confirmed that this difference could not be explained by bacterial multiplication, which started only 4–8 h after release (Figure 6I). These observations unambiguously demonstrate that dissemination is possible for all strains and therefore that IBC is not a dead end for AIECs.

## Discussion

### Convergent evolution of AIECs leads to IBC formation

Alterations in the composition and function of gut microbial communities are implicated in inflammatory bowel disease (IBD), with Crohn's disease (CD) patient microbiomes showing an increase in mucosa-associated Enterobacteriaceae, including AIECs.^9^^,^^46^ AIEC are defined by their *in vitro* adherent-invasive behavior. There is a clear need to extend our knowledge of AIEC to pathoadaptive determinants of host colonization and virulence. Our study of 13 new AIEC strains confirmed previous results, indicating that the AIEC phenotype encompasses *E. coli* strains with very different genome contents.^16^^,^^47^^,^^48^ This genomic diversity is attributed to both SNP polymorphisms of core genes (Figure 1C) and horizontal gene transfer (Figure 1B). The AIECs in our collection belong to the A, B1, B2, and D phylogroups. Despite this diversity, all the tested AIECs exhibited a common phenotype: the formation of intracellular bacterial communities (IBC) within Lamp-I-positive compartments inside macrophages. These IBCs differ significantly from those of *Salmonella*, *Shigella*, or uropathogenic *E. coli* (UPEC) observed under the same conditions (Figure 1). To refine this genomic-phenotypic analysis, we assembled and annotated the genomes of four new AIEC strains (CEA106S, CEA212U, CEA224S, and CEA601S). Compared to LF82, each of these strains carried more than 350 specific genes, with only 31 genes shared among the four and LF82 but absent from a commensal control (Supplementary Figure S3 and Supplementary Text). The genomes of AIECs are particularly enriched in prophages, which may encode morons^37^ Supplementary Table S2), and they also contain conjugative plasmids that carry virulence factors such as T3SS in CEA224S. Each studied AIEC harbor a distinct set of putative virulence factors, including siderophores, adhesins, effectors, toxins, and secreted proteases (Supplementary Figure S3). This rich genomic diversity may result from fortuitous evolution^49^ and various selective pressures that AIECs encounter in their diverse niches.^50^^,^^51^ Therefore, we propose that convergent evolution leads to the IBC phenotype. These findings suggest that in CD patients, macrophages or other immune cells impose selective pressure on mucosa-associated *Enterobacteriaceae*. Supporting this hypothesis, the colonization of *lamina propria*'s immune cells by *Proteobacteria* during CD^7^ and the strong positive correlation between AIEC replication in macrophages and the severity of both macroscopic and microscopic lesions in mice^5^ have been observed.

### IBC formation relies on different tactics

**Figure 7.**
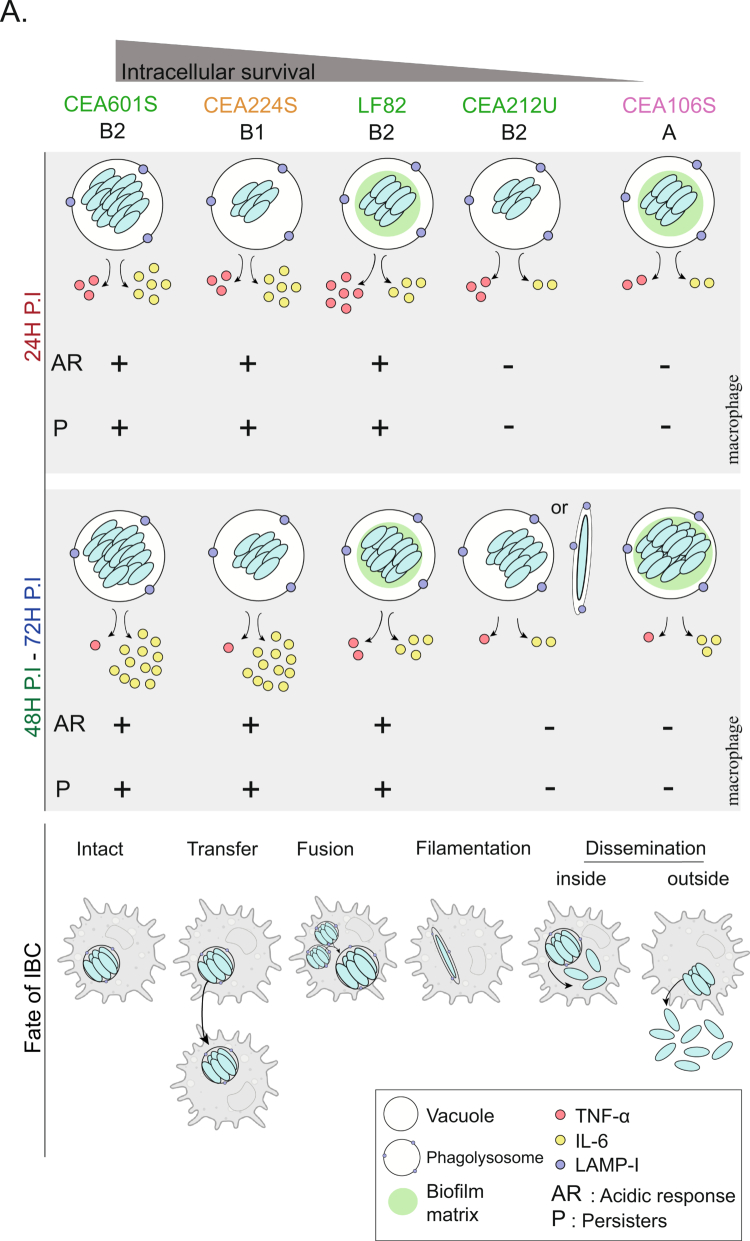
IBC formation relies on different strategies. A) Overview of the various survival, multiplication and dissemination strategies employed by the five AIEC strains studied and their consequences for the macrophage inflammatory response.

We observed that AIECs rely on different tactics to persist and multiply as IBC within macrophages. The five tested strains were grouped into three categories (Figure 7; Supplementary Table S5).

**LF82, CEA601S and CEA224S:** These three strains display the best survival rate at early and late time points after phagocytosis (Figure 2C). Their IBCs grow during the first 48 h in an acidic phagolysosomes, and they continuously produce more persisters. This pattern follows the earlier characterization of LF82 intra-macrophage persistence and multiplication.^12^^,^^13^ The macrophage infected by these three strains exhibit a strong inflammatory response and a reduction in their lysosome biogenesis capacity. However, in this group, only LF82 exhibited intense WGA staining (Supplementary Figure 9), suggesting that a robust adhesive matrix is not required for IBC formation and bacterial survival under these conditions. This might also be the case for IBC formed by UPEC in bladder cells.^52^

**CEA106S:** At first glance, our CFUs and IBC counting data suggested that CEA106S is the less efficient AIEC of our group for survival within macrophages (Figure 2C). These observations might actually be misleading because the ejection of bacteria outside of macrophages is very frequent with this strain. Bacteria grow efficiently (displaying a high frequency of bacteria with FtsZ-ring, important EPS staining, and a low amount of persisters), they form large IBC, that reach carrying capacity and burst, releasing live bacteria in the medium that are not counted by a classical gentamicin protection assay. In good agreement with the hypothesis of efficient multiplication, we observed that although inflammation and autophagy were activated during CEA106S infection, the bacteria stopped rapidly the induction of its acid stress response (Figure 4A and Supplementary Figure S7C). Lysosome and membrane biogenesis capacities are restored compared to other AIECs and eventually enhanced compared to non-infected macrophages. Altogether, these observations suggested that CEA106S is able to detoxify its vacuole (Figure 4A and Figure 4C) and presumably to impose feedback on the macrophage transcriptome to produce more building blocks for their phagolysosomes (Figure 3G,H). Considering its filiation with the A phylogroup, where a majority of commensal *E. coli* was notable.

**CEA212U:** CEA212U belongs to the canonical B2 phylogroup; however, it behaves closely to CEA106S from the A phylogroup. It did not produce high levels of CFU at 48 and 72 h (Figure 2C), did not induce acid stress and did not accumulate persisters (Figure 4A and 4B). Differently from CEA106S, CEA212U induces a reduced inflammatory response with lower expression of most chemokines compared to other AIECs. Strikingly, it elongates, forming large filaments that may completely resume cytokinesis. These findings suggest that these genes are metabolically active. Although the filamentation phenotype recalls from UPEC dissemination strategy, we do not think that CEA212U uses it as a dissemination strategy. Bacterial ejection from dying macrophage is only observed for IBC containing small cells (Figure 6). Since CEA212U lacks about 100 genes that are present in all other members of the B2 group (LF82, AIEC17, CEA601S and CEA304S) and also exhibits many SNPs (Figure 1B,C), it is possible that its original survival strategy reflects a partial loss of function. This is to be taken into consideration when interpreting results from UM146, an emerging AIEC model phylogenetically close to CEA212U.^5^

### Dissemination of AIECs

Earlier studies on macrophage infection by LF82 showed that it neither escapes from the phagolysosome nor induces macrophage death.^9^ Consistently, RNA-seq analysis indicated that LF82 induces anti-apoptotic functions in macrophages (Figure 3).^13^ This led to the consensus that AIEC replicates within macrophages without causing host cell death.^53^ However, the results presented sharply contradict this view (Figure 6). Our findings indicate that while AIECs can kill the macrophage and be ejected synchronously into the external medium, they also exploit additional dissemination strategies. Some strains break the vacuolar membrane and disseminate into the cytosol, whereas others disseminate as an intracellular bacterial community (IBC) that can be recaptured by neighboring macrophages, subsequently fusing with their endogenous IBC. Additionally, we observed events similar to the vomocytosis process, a transfer of loaded phagolysosomes from a live macrophage to a new macrophage, a phenomenon described for fungal pathogens.^54^ Notably, the frequency of these dissemination processes in the population of infected macrophages varies from one strain to another. We postulate that these differences may reflect specific macrophage‒pathogen interactions. Further studies are needed to test whether these dissemination processes are relevant for AIEC-infected tissues within *in vivo* models and CD.

### Could IBC characteristics be used as a marker of AIEC pathogenicity?

The search for genomic markers specific to the AIEC phenotype was unsuccessful. Given that bacterial survival within immune cells has been identified as a marker of Crohn's disease (CD)^7^ and as an indicator of macroscopic and microscopic intestinal lesions in mice,^5^ we hypothesized that an in-depth analysis of the phenotypes associated with AIEC survival within macrophages would help classify them. First, we demonstrated that all AIECs, unlike *Salmonella*, *Shigella*, UPEC, or commensals, form IBC. Therefore, the presence of IBCs inside macrophages differentiates AIECs from other enterobacteria but does not distinguish among different AIEC strains. The analysis of more than 60 phenotypic characteristics of AIEC infection allowed us to identify several interconnected features. For instance, intracellular survival, persister frequency, vacuole acidification, and IL-6 production were highly correlated. In contrast, the WGA labeling of a putative extracellular matrix, which appeared to reinforce LF82 survival,^13^ did not correlate with the intracellular survival of other strains. These findings suggest that successful AIECs, those with higher survival rates, rely more on their ability to cope with macrophage responses rather than on their ability to build a protective matrix. This observation generalizes to the AIEC group's previous findings showing that acidification and inflammation play key roles in LF82 multiplication.^11^ We add to this scenario the significant contribution of continuous persister production during growth under stress. Although additional validations with more strains and *in vivo* assays are required, we propose a minimal model for optimal AIEC multiplication. It requires phagolysosomal activation for multiplication and simultaneous persister formation. IBCs formed in this toxic environment accumulate induced persisters that tolerate stress, while replicating bacteria are at risk, and their debris stimulates macrophage signaling, including IL-6 production. This cytokine promotes the recruitment of additional immune cells to the infection site, potentially facilitating the dissemination of AIECs to other immune cells (Figure 6D,E). Additional options, such as extracellular matrix production or the secretion of effectors by T3SS might confer additional advantages to certain strains. Alternatively, others may select a different path involving phagolysosome detoxification, which appears less efficient, possibly because it does not involve IL-6 production. IL-6 imbalance has been documented in CD, and it is proposed that it favors an intestinal microenvironment prone to chronic inflammation. Although further *in vivo* studies will be required, these observations open the possibility that targeting IL-6 could be part of therapeutic strategies.^55^

## Supplementary Material

Supplementary Material Supplementary-Figures-GM.pdf

Supplementary Material Table_S1.xlsx

Supplementary Material Table_S2.xlsx

Supplementary Material Table_S3.xlsx

Supplementary Material Table_S4.xlsx

## Data Availability

Genomes of strains CEA212U (GCF_051122395.1), CEA601S (GCF_051122395.1), CEA106S (GCF_051122425.1) and CEA224S (GCF_051122395.1) are available at NCBI. The RNA-seq data are available at GEO GSE307944. Numerical data are available on Figshare with the DOI/10.6084/m9.figshare.30113062. The microscopy and flow cytometry data that support the findings of this study are available from the corresponding author, [OE], upon reasonable request.
