# Supplementary material for: Persistence mechanisms of Crohn's disease-associated adherent invasive Escherichia coli within macrophages
Source: Gut Microbes. 2025 Nov 25;17(1):2587402. doi: 10.1080/19490976.2025.2587402 (PMC12931692; doi:10.1080/19490976.2025.2587402)
Supplement: Supplementary Material — Supplementary-Figures-GM.pdf [file KGMI_A_2587402_SM8264.pdf]

## Supplementary Materials

### Supplementary text: Annotation of the virulence factors harbored by the five AIEC strains

#### *Secretion*

The secretion of virulence factors has been extensively shown to influence the colonization of host cells by intracellular pathogens (Poirier V & Av-Gay Y, 2015). Research on pathogen models such as *Salmonella*, *Shigella*, and *Yersinia* has elucidated the essential roles played by Type 3 secretion systems (T3SS). In addition to the general Type 2 secretion system, the LF82 genome exhibits two complete and one partial Type 6 secretion systems (T6SS), a T4SS, and numerous autotransporters (T5SS), but lacks a T3SS (Fig. 2B and Supplementary Fig. S3B). However, the involvement of these systems in the colonization of epithelial cells or macrophages by LF82 has not yet been observed.

Regarding secretion, strain CEA224S (B1 phylogroup) stands out as the most distinctive, as it harbours a complete type III secretion system on its large plasmid, suggesting its capability to secrete virulence factors into its host (Supplementary Fig. S3B). Furthermore, it also carries a partial type III secretion system similar to the PrgK, PrgI, and OrgB proteins located between the *yqeG* and *ygeR* genes on the chromosome (Supplementary Table S2). This cluster is also present in strain CEA106S (A phylogroup) at the same position. LF82 exhibits two complete type VI secretion systems, defined as pathogenicity islands PAI-I and PAI-III (Miquel S et al., 2010). Strains CEA212U, CEA224S, and CEA106S also contain a T6SS at the PAI-I location, while strain CEA601S harbours a large gene cluster encoding sugar metabolism enzymes, transporters, and transcription factors in this position. Strains CEA212U and CEA224S also display putative T6SS at the PAI-III locus, whereas this locus is empty in strains CEA601S and CEA106S. All strains exhibit a wide array of putative autotransporters (T5SS) and one or two T4SS frequently found on their respective plasmids (Supplementary Fig. S3B).

#### *Appendages*

AIECs exhibit various appendages that may facilitate motility, adherence, invasion, and protection from phagocytosis (Fig. 2B). Motility is mediated by the flagellum, which is present in every strain. Adherence is facilitated by fimbriae, which can vary in type. All strains possess the operon necessary for the synthesis of type 1 fimbriae (*fimA-H*). Additionally, LF82 harbors a gene cluster for long polar fimbriae, while CEA601S and CEA106S feature afimbrial adhesins (*afaABCD*), CEA212U contains a P fimbriae gene cluster (*papA-G*), and CEA224S possesses both adhesive fimbriae (*faeC-J*) and long polar fimbriae (Supplementary Fig. S3A). Each AIEC strain encodes capsule synthesis gene clusters. In LF82, capsule synthesis is enabled by the PAI-IV. This locus exhibits high polymorphism; it consistently contains genes involved in capsule synthesis and a putative secretion system as in LF82, but may also

contain genes for the P fimbriae gene cluster (*papA-G* operon), phosphoglycerate transport (*pgtP-pgtCBA*), or virulence factors (*sat* protease, colicin, *espK*) in other strains. The *E. coli* common pilus (ECP) gene cluster is present in all strains, promoting binding to the extracellular matrix (Mondal R et al., 2022); CEA212U and CEA106S additionally display FdeC, a homolog of Invasin and Intimin, which facilitates kidney and bladder colonization by UPEC (Nesta B et al., 2012). Other invasion factors such as Tia/Hek, IbeABC (Cieza RJ et al., 2015), and Hra were detected in these strains.

### *Iron capture*

Iron acquisition is essential for bacteria as a nutritional requirement. As part of the nutritional immunity response host secrete sequestration proteins to deplete free iron and limit pathogen growth (Ullah I & Lang M, 2023). AIEC genomes contain more iron capture systems compared to other *E. coli* (Dogan B et al., 2014). We demonstrated that activation of the Yersiniabactin production island HPI (PAI-II) in LF82 corresponds to the beginning of bacterial multiplication within macrophages. This prompt us to investigate the distribution of iron capture systems in our bacterial cohort (Supplementary Fig. S3E). The PAI-II locus is occupied by the HPI in LF82, CEA601S, CEA212U and CEA106S but empty in CEA224S. Strains from the B2 group also display the Chu haeme capture system.

### *Virulence effectors*

The genomes of AIEC encode several potential offensive virulence factors (Supplementary Fig. S3A). These include Vat and Hbp proteases in LF82, CEA601S, and CEA212U; Colicins in CEA212U; and Sat and SenB toxins in CEA106S. Strains CEA224S, which possesses a putative T3SS, also carry the same group of secreted factors: EspX5, EspX1, EspR1, EspX4, EspL4, and EspY1. Among these, only EspY1 has known host cell targets related to apoptosis or cell cycle regulation.

This diverse arsenal suggests that these bacteria may react differently in various host environments to colonize tissues or cells. This highlights the importance of conducting thorough phenotypic analyses before designing any potential therapeutic strategies. Furthermore, this necessity is underscored by the significant antibiotic resistance potential observed in our AIEC cohort (Supplementary Fig. S3C).

## Legend of the Supplementary Figures

### Supplementary Figure 1

A) Adhesion assay of AIEC on epithelial cells (top) and invasion assay of AIEC into epithelial cells (bottom). B) Imaging of IBC within Lamp-I (in grey) positive vacuole in THP-1 macrophages by AIECs (in red). The scale bars are 5 µm C) For each time point in the kinetic study, a summary sheet shows the percentage of THP-1 macrophages that are non-infected, infected with IBCs, or harboring isolated bacteria. Additionally, a violin plot illustrates the number of bacteria per IBC, with the median represented by a full line and the quartiles by dashed lines. Data were analyzed using the Mann-Whitney test, with significance indicated as follows: *P < 0.05, **P < 0.01, ***P < 0.001, ****P < 0.0001.

### Supplementary Figure 2

A) Plasmids of the five AIEC strains visualized on an agarose gel. B) Table summarizing the different AIEC plasmids, including their sizes, sequencing depths (a proxy for copy number), and replication types. The coding sequences (CDS) predicted for plasmids pLF82, CEA106Spa.1, CEA106Spb.1, CEA106Spc.1, CEA212Up.1, CEA224Sp.1, and CEA601Sp.1 are detailed in Table S2. C) Circos plot comparing the gene content of the different plasmids of the 5 AIEC and that of pNRG857C (NRG857C plasmid; reference AIEC) with pUM146 (UM146 plasmid; reference AIEC)

### Supplementary Figure 3

Presence/absence matrix of genes for the five AIEC strains and MG1655 K12. The colour code is consistent across all graphs: green squares indicate the presence of a gene in the chromosome, while black squares denote its absence. Red squares signify the presence of the gene on a plasmid. For systems with multiple genes, light green squares represent partial presence. A) Matrix of predicted virulence factors based on VFDB. B) Matrix of predicted secretion systems identified using MacSyFinder on the Mage Genoscope website. C) Matrix of predicted antimicrobial resistance based on AMRfinder. D) Matrix of predicted prophages using Phigaroin available on the Mage Genoscope website. E) Matrix of iron acquisition systems (manually curated). F) Matrix of toxin-antitoxin system predicted by TAfinder2.0. H) Venn Diagram of the genes present in the five AIECs excluding the *E. coli* core genome based on *E. coli* K12 gene content. G) Matrix of defense system predicted by CRISPRCasFinder on the Mage Genoscope website. H) Only 31 genes are present in each of these five AIEC strains but absent from the *E. coli* K12 genome. This analysis was performed on the Mage Genoscope website. I) Map of the T3SS harboured by the plasmid of CEA224S in comparison with that of *Salmonella Enterica*. J) Intracellular ratio rate between 24-hour P.I and 1-hour P.I of CEA224S and CEA224S ΔT3SS. P-values are indicated (Student’s t-test: *P < 0.05, **P < 0.01, ***P < 0.001, ****P <

0.0001); line in the box represents to the median, and whiskers indicate the minimum and the maximum values; n ≥ 3.

**Supplementary Figure 4**

A) KEGG analysis of the macrophage's pathways that are up regulated by the persistence of AIEC compared to N.I. macrophages. B) Principal component analysis of the expression of a subset of genes coding for chemokines. C) Examples of genes exhibiting noticeable up regulation during AIEC persistence.

**Supplementary Figure 5**

A) KEGG analysis of the macrophage's pathways that are down regulated by the persistence of AIEC compared to N.I. macrophages. B) Principal component analysis of the expression of a subset of genes involved in lysosome biogenesis. C) Examples of genes exhibiting noticeable down regulation associated with AIEC persistence.

**Supplementary Figure 6**

KEGG analysis of the macrophage's pathways that are up (left) or down (right) regulated by the infection with the indicated AIEC strain compared to LF82.

**Supplementary Figure 7**

A) In vitro validation of the *asr* reporter (pSM1690 P*asr*-GFP-AAV). LF82 culture were shifted from pH 7.4 to pH 4.7 (left) or shifted from pH 4.7 to pH 7.4 (right). Upon pH drop, the GFP-AAV production was maximal in less than 1 hour and then stayed above detection limits for ~200 min. Upon pH neutralization, GFP-AAV dropped to undetectable levels in 120 min. These results suggest that the *asr* reporter accurately monitors acidification of the bacterial environment in a two hours window. B) Histogram from FACS analysis of the *asr* reporter at 1, 24, and 48 hours P.I. C) qRT-PCR analysis of the induction of the AIEC acid stress response (*asr*) at 24 hours post-phagocytosis (P.P.) in THP-1 macrophages. The ratio of *asr* expression levels in stationary-phase cultures in rich liquid medium versus macrophages was calculated. The data represent the relative quantification of *asr*, normalized to the *rpsM* reference gene. P-values are indicated (Mann-Whitney test: $P < 0.05$, *$P < 0.01$, **$P < 0.001$, ***$P < 0.0001$). Error bars represent the mean and standard deviation (SD); n ≥ 3. E) Timelapse imaging of LF82 pSM1690 P*asr*-GFP in the absence or presence of BAF A1 to curb macrophage autophagic response.

**Supplementary Figure 8**

A) Flow cytometry quantification of AIEC response to intracellular nutrient scarcity (*rrnBP1*). P-values are indicated (Student's t-test: *P < 0.05, **P < 0.01, ***P < 0.001, ****P < 0.0001); error bars

represent the mean and standard error of the mean (SEM); n ≥ 3. Flow cytometry gating strategies are described in Supplementary Figure 8. B) Imaging of AIEC strains containing an FtsZ-GFP reporter within Lamp-1 vacuoles (grey) in THP-1 macrophages at 24 hours post-phagocytosis, P.P. (left panel) and 48 hours P.I. (right panel). Scale bars represent 20 µm. C) Quantification of FtsZ-positive bacteria within IBCs at 24 and 48 hours P.I. Th violin plots represent the distribution of the result for each time point, with the red line indicating the median, and the black line marking the quartiles. D) Imaging of filamentous CEA212U bacteria within THP-1 macrophages at 48 hours (top panel) and 72 hours (bottom panel) post-phagocytosis (P.P.). Bacteria are shown in red, and Lamp-1 vacuoles are labelled in grey. Scale bars represent 5 µm. E) Timelapse of CEA212U on LB-Agar pad, with 20-minute intervals between each image. Scale bars represent 10 µm.

**Supplementary Figure 9**

A) Confocal images of IBCs formed by AIEC (in red), showing WGA lectin-labelled polysaccharides (in cyan) and phagolysosomes marked by Lamp-I antibody (in grey). White arrows indicate WGA labelling, suggesting the presence of an intraphagosomal exopolysaccharide matrix. Scale bars represent 5 µm. B) STED imaging of IBCs within THP-1 macrophages at 24 hours P.I. Bacteria are shown in blue, exopolysaccharide (EPS) matrix is labelled with WGA lectin in red, and phagolysosomes are labelled with Lamp-I antibody in grey. On the right, a zoom on the WGA labelling. Scale bars represent 5 µm. C) FRAP experiments on phagolysosomes containing LF82-GFP, CEA601S-GFP, or CEA106S-GFP, conducted at 48 hours post-phagocytosis (P.P.). Data were normalized to value obtained immediately after photobleaching to assess fluorescence recovery. On the right is a representation of an IBC (grey) before bleaching, just after the bleaching and at the end of the recovery period (155 min). The red circles indicate the bleached area. Scale bars represent 5µm.

**Supplementary Figure 10**

A) Table of the p-values obtained for the experiments presented on Figure 4 and supplementary figure 6 for persisters (left panel), *asr* FACS analysis (center panel), and *rrnBP1* (right panel). Data were analyzed using Student's t-test: *P < 0.05, **P < 0.01, ***P < 0.001, ****P < 0.0001. B) Proportion of viable bacteria during the infection kinetics in THP-1 macrophages compared to levels at 1 hour post-phagocytosis (P.P.). Each graph represents a single strain. P-values are indicated (Mann-Whitney test: *P < 0.05, **P < 0.01, ***P < 0.001, ****P < 0.0001). C) Proportion of viable bacteria in THP-1 macrophages at different time points compared to 1 hour P.I. Each graph represents a different time point. P-values are indicated (Mann-Whitney test: *P < 0.05, **P < 0.01, ***P < 0.001, ****P < 0.0001).

Figure Supplementary 1

A.

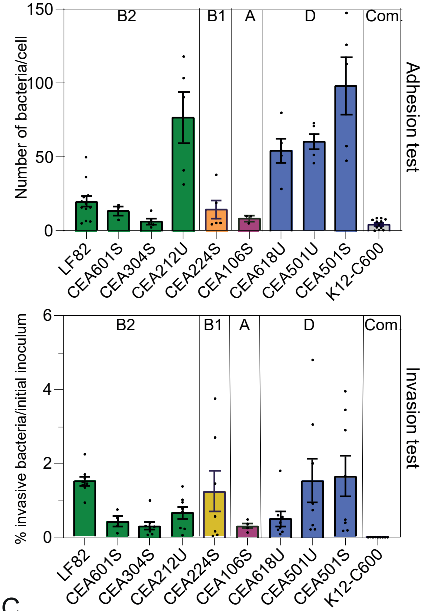


B.

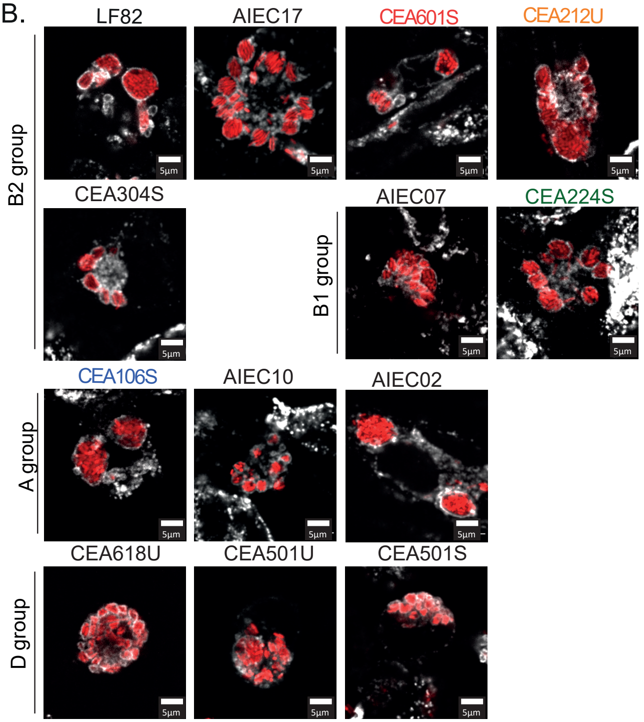


C.

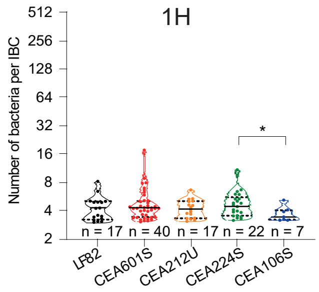


1H

| | LF82 | CEA601S | CEA212U | CEA224S | CEA106S |
|---|---|---|---|---|---|
| Non-infected THP1 (%) | 15 | 38 | 54 | 12 | 52 |
| THP-1 with IBC (%) | 8 | 8 | 5 | 12 | 7 |
| THP-1 with isolated bacteria (%) | 77 | 55 | 39 | 76 | 40 |

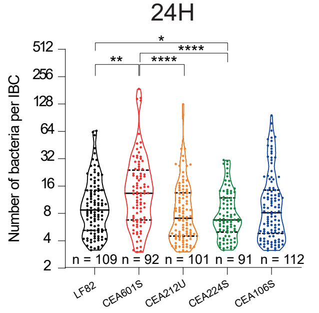


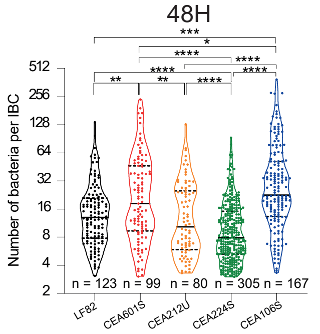


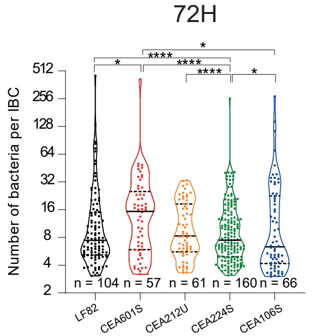


24H

| | LF82 | CEA601S | CEA212U | CEA224S | CEA106S |
|---|---|---|---|---|---|
| Non-infected THP1 (%) | 22 | 50 | 61 | 54 | 50 |
| THP-1 with IBC (%) | 26 | 11 | 7 | 5 | 17 |
| THP-1 with isolated bacteria (%) | 52 | 39 | 32 | 41 | 33 |

48H

| | LF82 | CEA601S | CEA212U | CEA224S | CEA106S |
|---|---|---|---|---|---|
| Non-infected THP1 (%) | 17 | 28 | 66 | 67 | 44 |
| THP-1 with IBC (%) | 22 | 14 | 4 | 5 | 43 |
| THP-1 with isolated bacteria (%) | 61 | 58 | 30 | 28 | 43 |

72H

| | LF82 | CEA601S | CEA212U | CEA224S | CEA106S |
|---|---|---|---|---|---|
| Non-infected THP1 (%) | 22 | 31 | 62 | 53 | 23 |
| THP-1 with IBC (%) | 9 | 9 | 6 | 13 | 11 |
| THP-1 with isolated bacteria (%) | 69 | 60 | 31 | 34 | 66 |

Figure Supplementary 2

A.

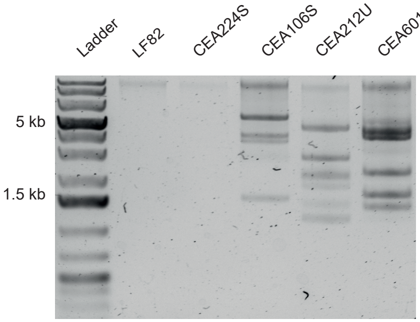


B.

| Strain | Size (bp) | Depth | Type / AMR gene |
|---|---|---|---|
| **LF82** | 108 379 | | |
| **CEA224S** | 133 924 | 2.35x | IncFII(pHN7A8) |
| **CEA106S** | 116 000 | 1.18x | Col156<br>*senB* (TieB enterotoxin) |
| CEA106S | 62 118 | 1.93x | |
| CEA106S | 42 251 | 3.18x | |
| CEA106S | 7 931 | 24.84x | |
| CEA106S | 5 139 | 56.47x | |
| CEA106S | 2 077 | 164.20x | Col(BS512) |
| **CEA212U** | 91 888 | 2.23x | IncB/O/K/Z<br>*aph(3'')-Ib, aph(6)-Id, blaTEM-1C, sul2 (ATB resistance)* |
| CEA212U | 6 647 | 45.02x | |
| CEA212U | 4 074 | 37.52x | |
| CEA212U | 2 671 | 79.81x | |
| CEA212U | 1 553 | 120.95x | |
| **CEA601S** | 61 246 | 3.16x | |
| CEA601S | 7 465 | 37.3x | |
| CEA601S | 6 342 | 52.05x | |
| CEA601S | 6 002 | 19.97x | |
| CEA601S | 5 631 | 94.50x | |
| CEA601S | 4 082 | 11.62x | |
| CEA601S | 3 187 | 154.74x | |
| CEA601S | 2 255 | 160.85x | |

C.

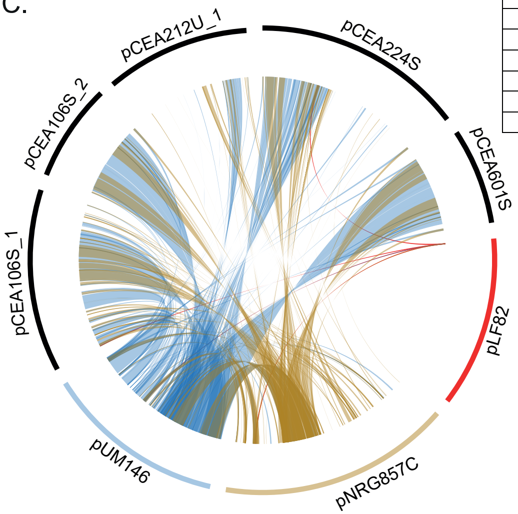

Figure Supplementary 3

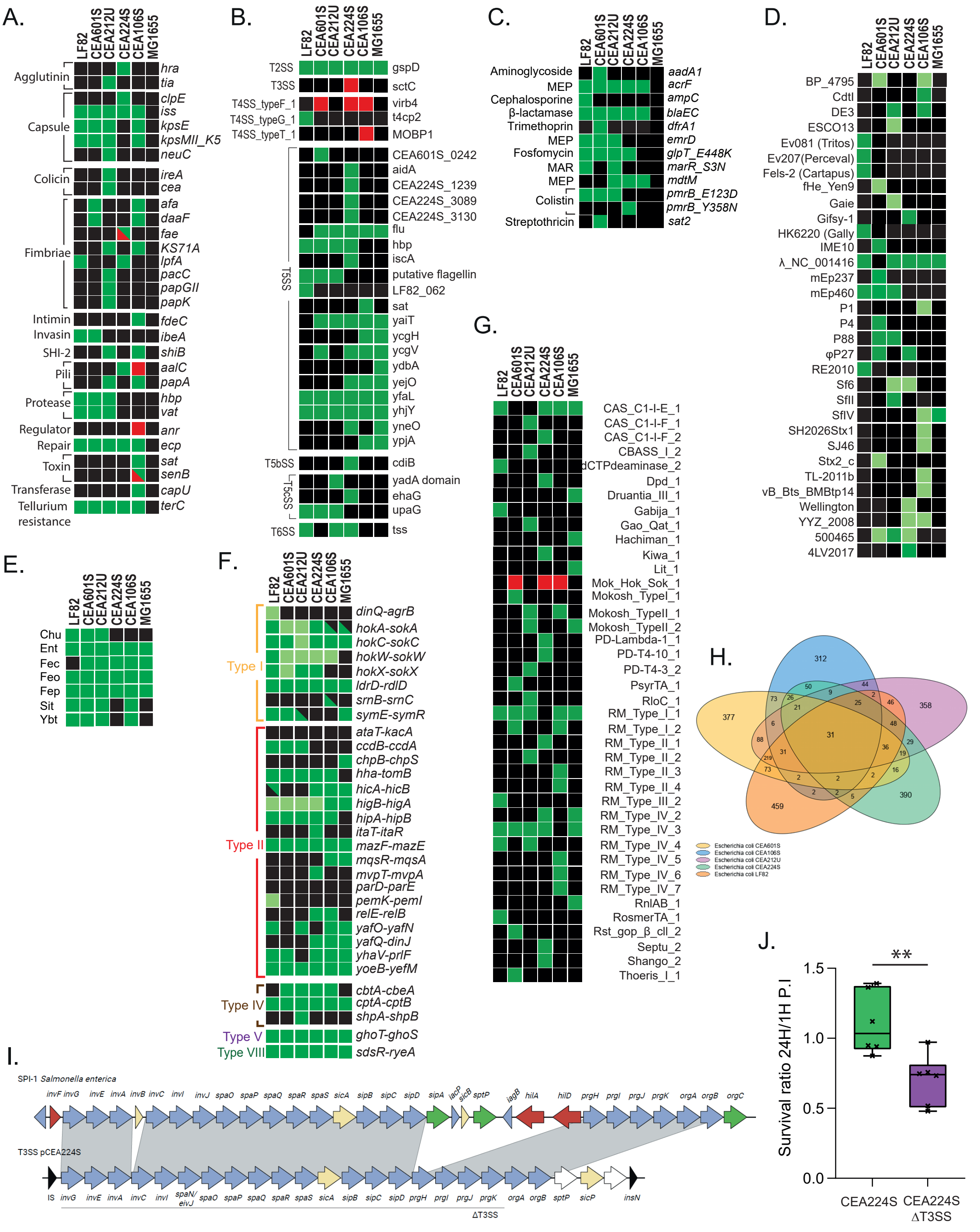

# Figure Supplementary 4

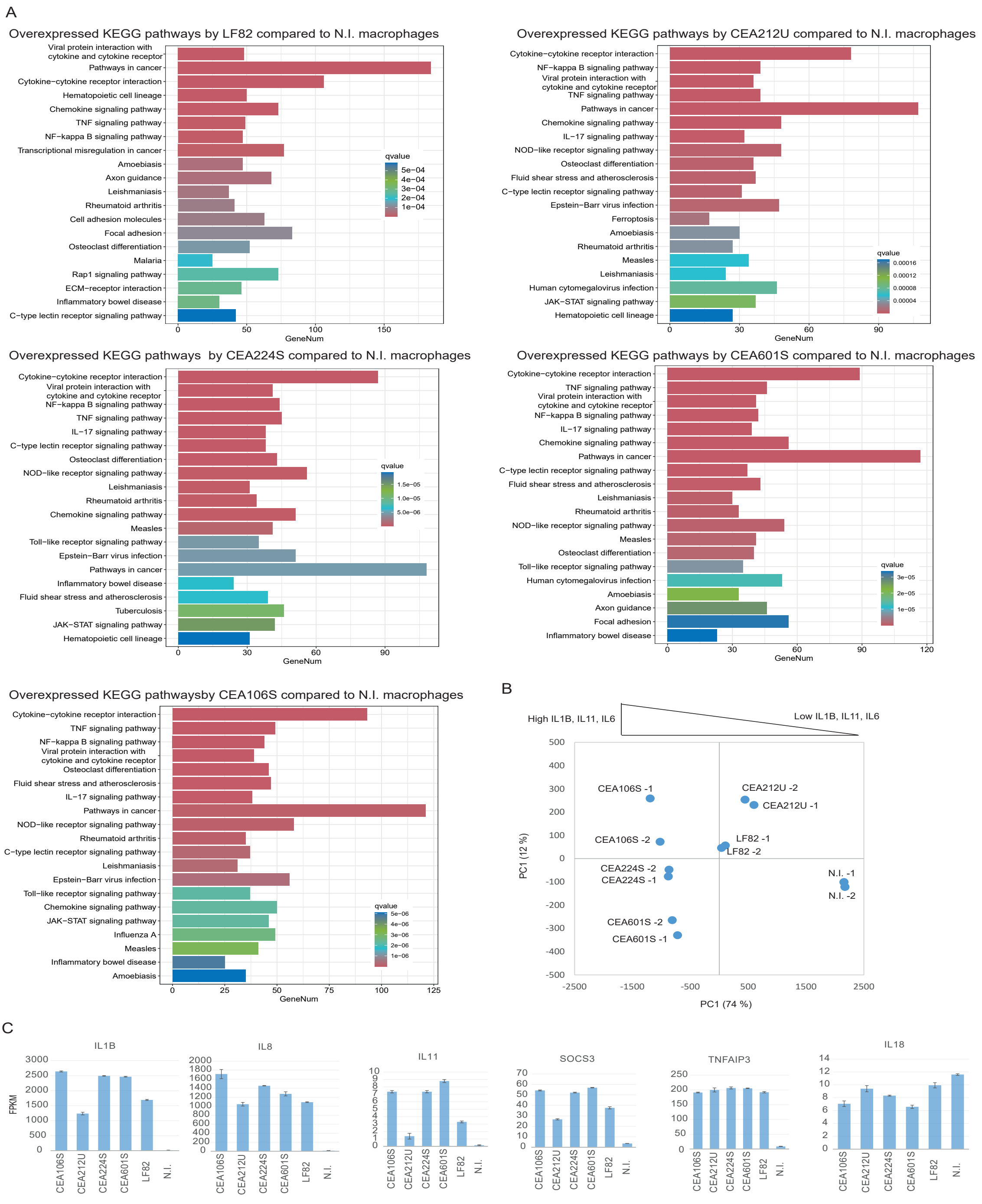

# Figure Supplementary 5

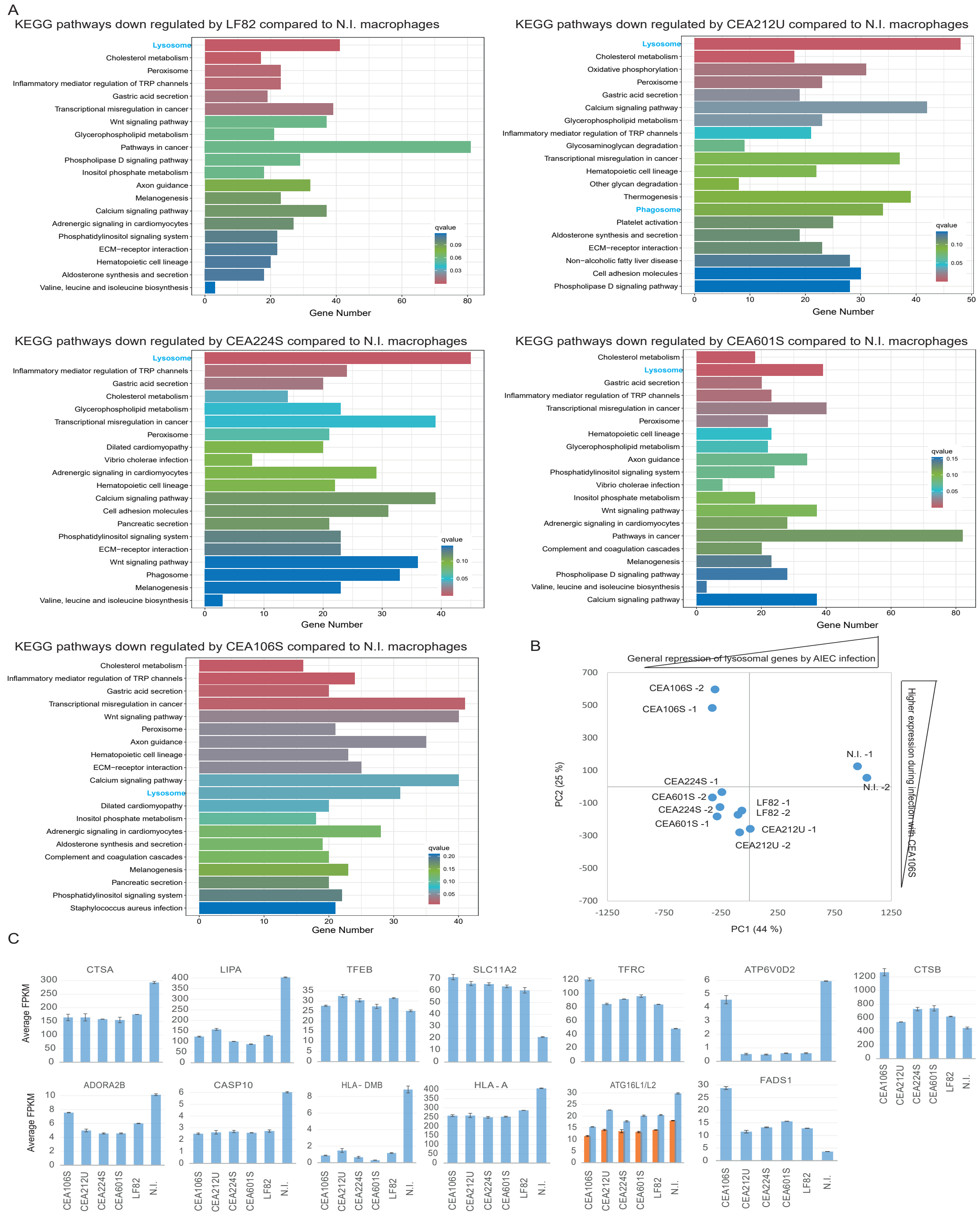

# Figure Supplementary 6

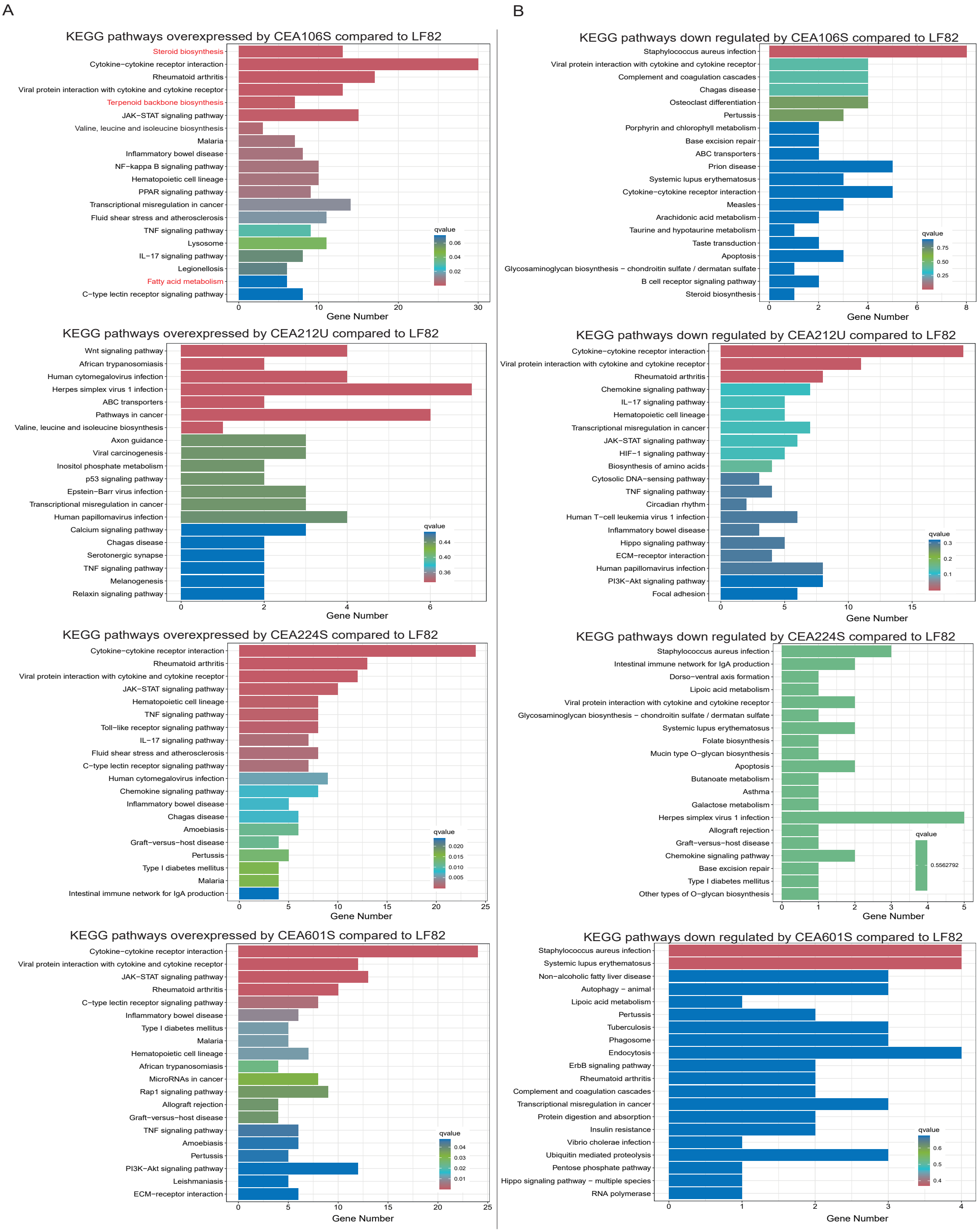

Figure Supplementary 7

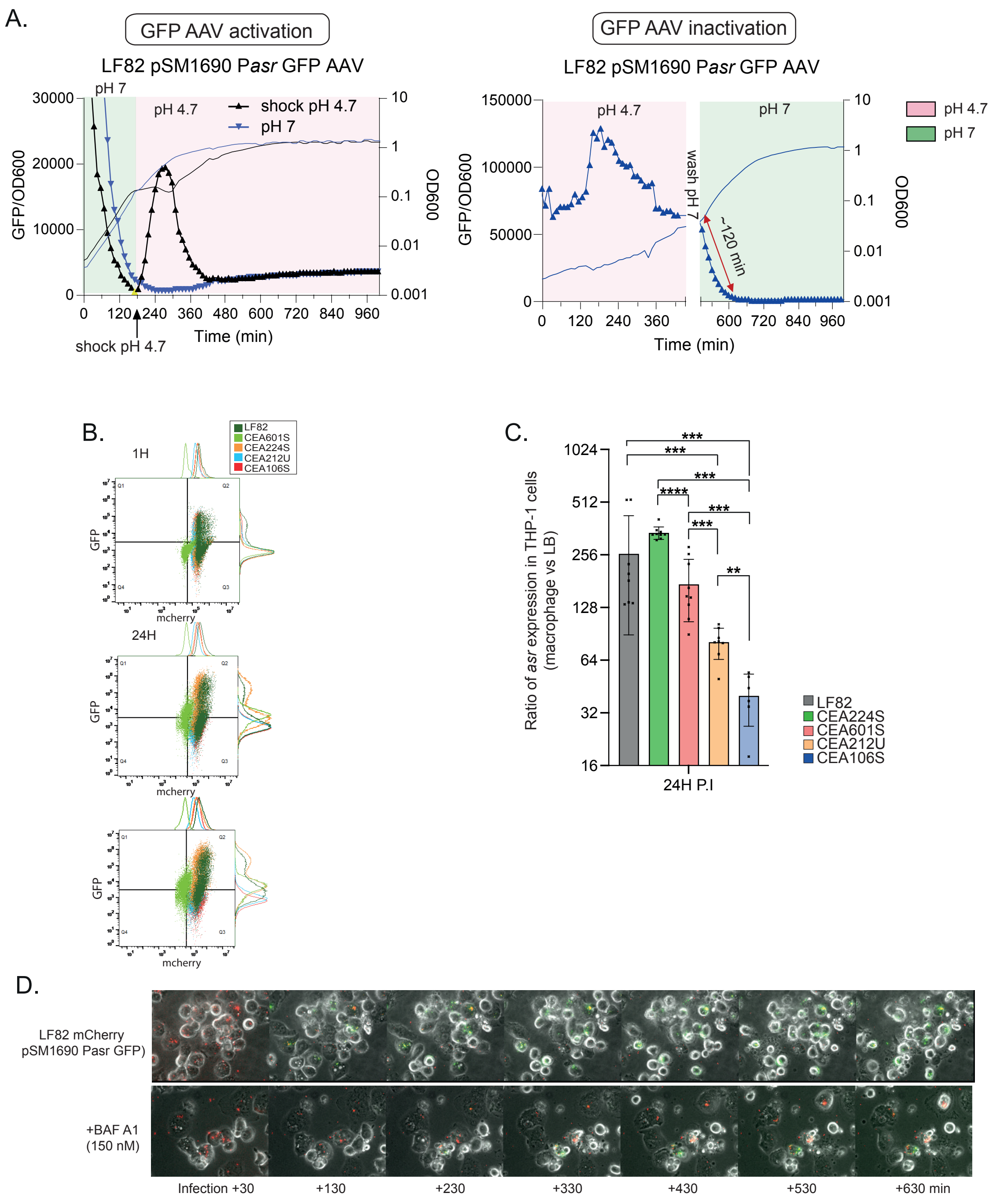

Figure Supplementary 8

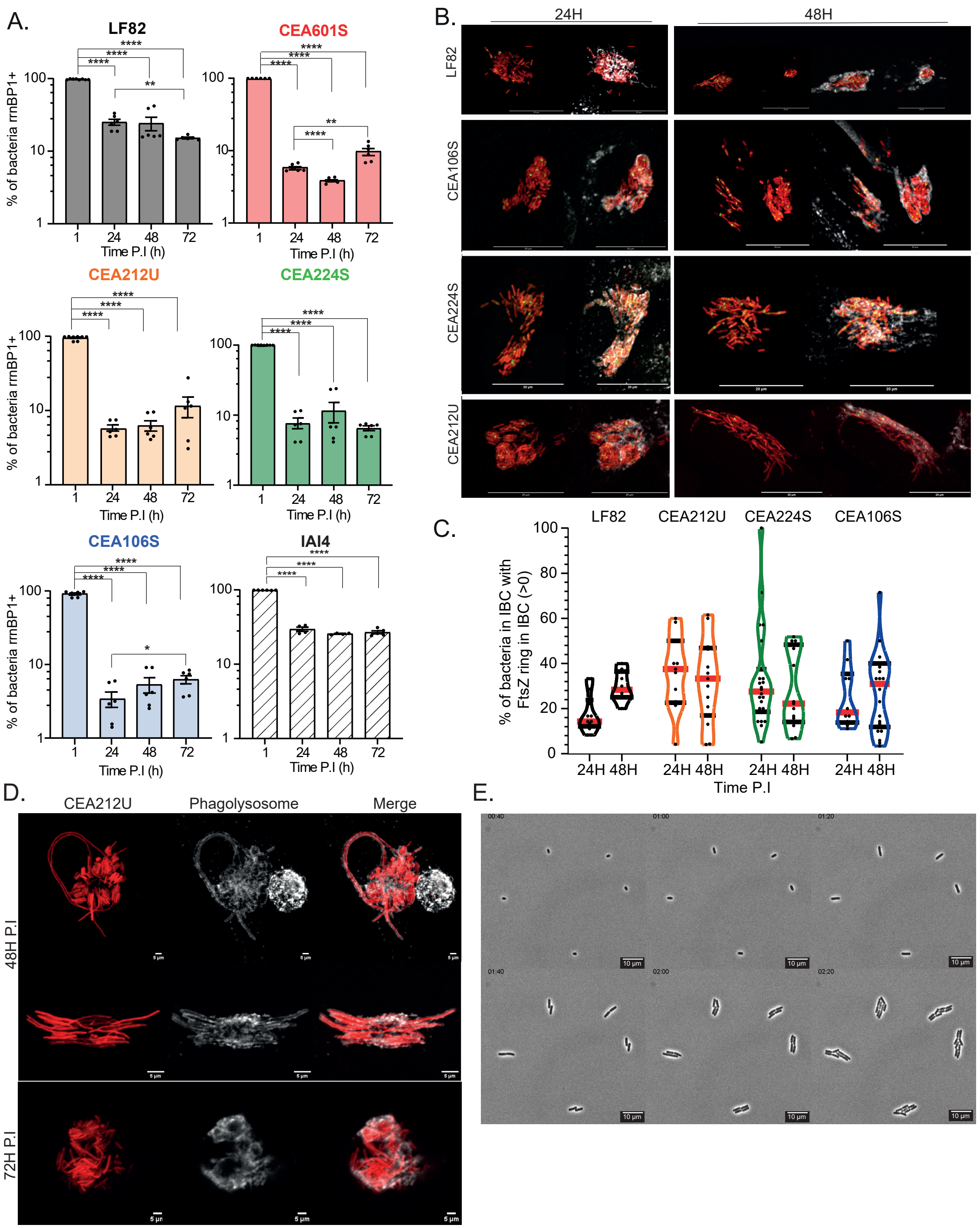

Figure Supplementary 9

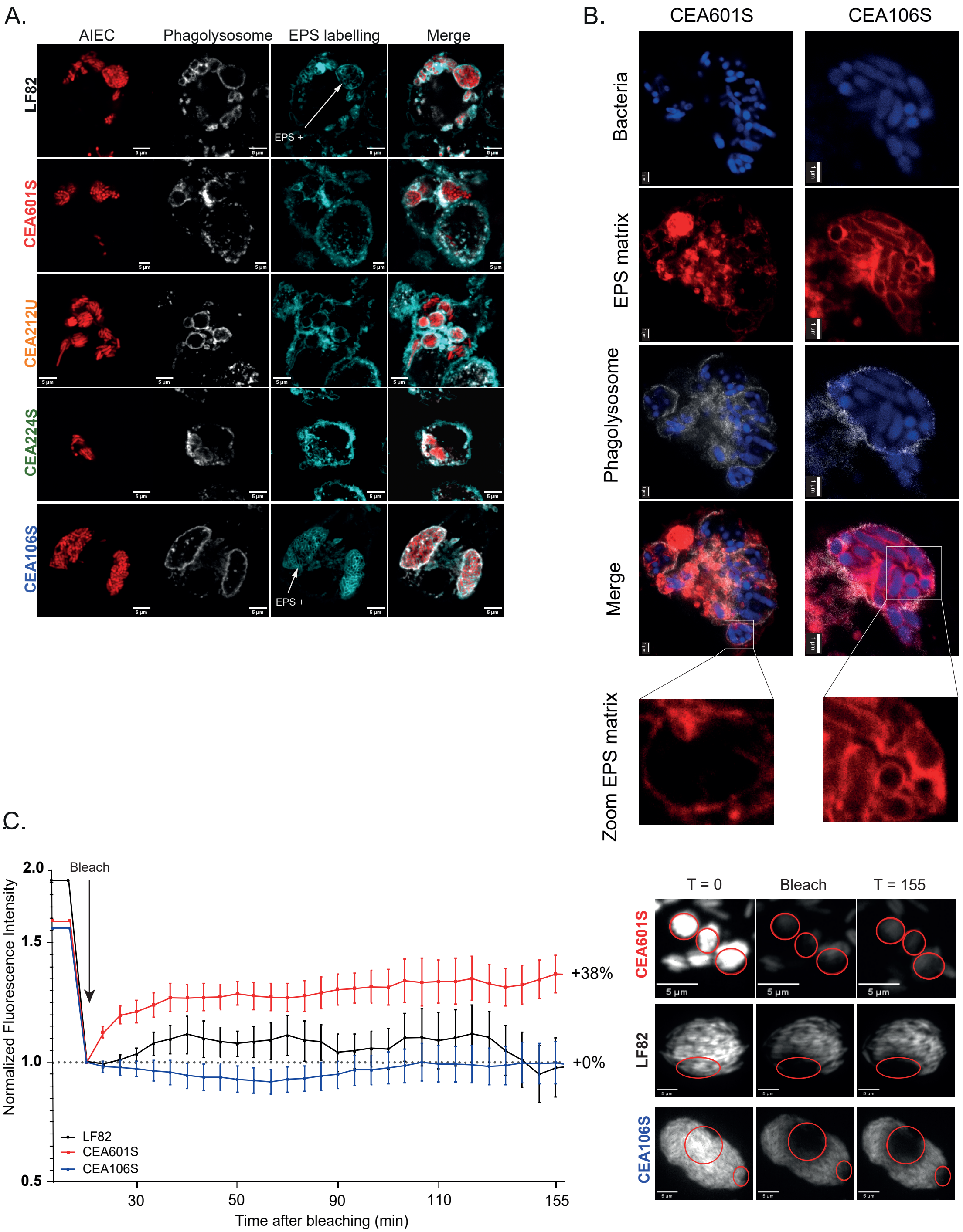

Figure Supplementary 10

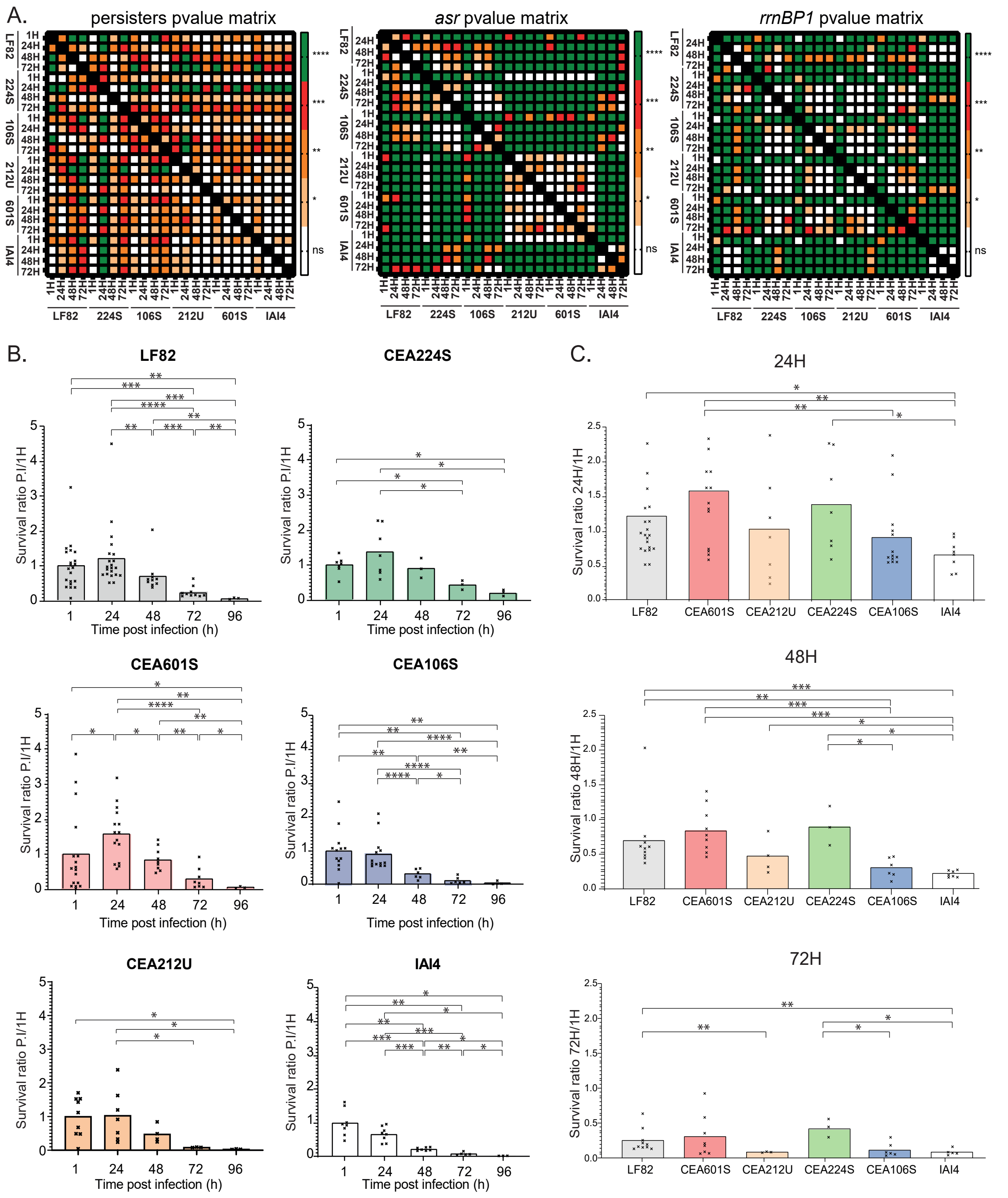

## Descriptions of the Supplementary Tables

### Table S1 – Genomic characteristics of the AIEC strains

This table provides an overview of the AIEC strains analyzed in this study. It includes the following information for each strain: strain name, genome length (bp), GC content (%), number of coding DNA sequences (CDS), phylogroup, serotype, NCBI BioProject identifier, NCBI BioSample number or NCBI reference number, NCBI accession number, and MicroScope assembly information (Excel file).

### Table S2 – Genome and plasmid annotation of the AIEC strains

This table contains the annotation of the genomes and plasmids of the AIEC strains. It includes general information on gene features, positions, predicted functions, protein products, classification, and various annotation metrics generated by the MicroScope pipeline (Excel file).

### Table S3 – RNA-seq analysis data

This table compiles the complete RNA-seq dataset generated in this study, including differential gene expression analyses, statistical outputs, and associated metadata (Excel file).

### Table S4 – List of bacterial strains used in the study

This table summarizes all bacterial strains used in the experiments described in the article. It corresponds to and complements the information provided in the *Materials and Methods* section.

### Table S5 – Summary of AIEC strategies

This table presents an overview of the different strategies used by the five tested AIEC strains to persist and multiply within macrophages.

Table Supplementary 5

| | Phylogroup | Intra-cellular biofilm | Acidic response | Persisters | IL-6 trend overtime | Macrophage's transcriptomic features | Characteristic phenotype |
|---|---|---|---|---|---|---|---|
| LF82 | B2 | Biofilm like (WGA and curli, csgD dependant) | yes | Frequent | medium | inflammatory and reduced lysosomal activity | Long term persistence as IBC |
| CEA601S | B2 | No WGA labelling and mobile bacteria inside IBC | yes | Frequent | high | Pro inflammatory and reduced lysosomal activity | Long term persistence as IBC |
| CEA212U | B2 | No WGA labelling | rare | Rare | low | M2 like inflammatory status and reduced lysosomal activity | Transient filamentation |
| CEA224S | B1 | Weak WGA labelling | yes | Frequent | high | Pro inflammatory and reduced lysosomal activity | Long term persistence as IBC with frequent fusions of IBC |
| CEA106S | A | WGA matrix, immobile bacteria inside IBC | rare | Rare | medium | Pro inflammatory, keep lysosomal activity, changes in lipid metabolism | Frequent vacuole rupture and bacterial ejection |

**Legend of the Supplementary Movies**

The movies described below are available on Figshare with the DOI: 10.6084/m9.figshare.30113062.

**Movie S1:** Cytokinesis of CEA212U filaments in a single macrophage. Imaging started at 48 hours P.I. and total movie length is 16h. The green channel corresponds to the projected maximum of all Z steps (40 x 0.2 µm).

**Movie S2**: Ejection of CEA106S through phagolytic vacuole explosion. Phase contrast (grey) and CEA106S (green). Imaging started at 48 hours P.I. and total movie length is 16h. The green channel corresponds to the projected maximum of all Z steps (40 x 0.2 µm). The grey channel corresponds to a single Z plane. The movie corresponds to the montage presented on Figure 4A.

**Movie S3**: Same as Movie S2 with only the bacteria represented. The movie corresponds to the montage presented on Figure 4A.

**Movie S4**: Dissemination of the strain CEA601S through phagolytic vacuole rupture within macrophage's cytoplasm. Phase contrast (grey) and CEA601S (green). Imaging started at 48 hours P.I. and total movie length is 16h. The green channel corresponds to the projected maximum of all Z steps (40 x 0.2 µm). The grey channel corresponds to a single Z plane. The movie corresponds to the montage presented on Figure 4B.

**Movie S5**: Same as Movie S4 with only the bacteria represented. The movie corresponds to the montage presented on Figure 4B.

**Movie S6**: IBC fusions observed for the strain CEA106S. Phase contrast (grey) and CEA106S (green). Imaging started at 48 hours P.I. and total movie length is 16h. The green channel corresponds to the projected maximum of all Z steps (40 x 0.2 µm). The grey channel corresponds to a single Z plane. The movie corresponds to the montage presented on Figure 4C.

**Movie S7**: Same as Movie S6 with only the bacteria represented. The movie corresponds to the montage presented on Figure 4C.

**Movie S8**: LF82 individual bacteria capture by a non-infected macrophage from an IBC formed in a different macrophage. Phase contrast (grey) and LF82 (green). Imaging started at 48 hours P.I. and total movie length is 16h. The green channel corresponds to the projected maximum of all Z steps (40 x 0.2 µm). The grey channel corresponds to a single Z plane. The movie corresponds to the montage presented on Figure 4D.

**Movie S9**: Same as Movie S8 with only the bacteria represented. The movie corresponds to the montage presented on Figure 4D.

**Movie S10**: Capture of CEA601S IBC from an IBC formed in a different macrophage. Phase contrast (grey) and CEA601S (green). Imaging started at 48 hours P.I. and total movie length is 16h. The green channel corresponds to the projected maximum of all Z steps (40 x 0.2 µm). The grey channel corresponds to a single Z plane. The movie corresponds to the montage presented on Figure 4E.

**Movie S11**: Same as Movie S10 with only the bacteria represented. The movie corresponds to the montage presented on Figure 4A.

**Movie S12**: Filamentation of the phagocyted CEA212U bacteria within phagolytic vacuoles. Phase contrast (grey) and CEA212U (green). Imaging started at 48 hours P.I. and total movie length is 16h. The green channel corresponds to the projected maximum of all Z steps (40 x 0.2 µm). The grey channel corresponds to a single Z plane. The movie corresponds to the montage presented on Figure 4F.

**Movie S13**: Same as Movie S12 with only the bacteria represented. The movie corresponds to the montage presented on Figure 4F.
